# [2]Biphenyl‐extended pillar[6]arene functionalized silver nanoparticles for catalysis and label‐free detection

**DOI:** 10.1002/smo.20230016

**Published:** 2023-11-14

**Authors:** Dongxia Li, Gengxin Wu, Xin Wang, Jia‐Rui Wu, Ying‐Wei Yang

**Affiliations:** ^1^ International Joint Research Laboratory of Nano‐Micro Architecture Chemistry College of Chemistry Jilin University Changchun China; ^2^ Key Laboratory of Automobile Materials of Ministry of Education and School of Materials Science and Engineering Jilin University Changchun China

**Keywords:** catalysis, host‐guest chemistry, macrocyclic chemistry, organic‐inorganic hybrid nanomaterials, supramolecular chemistry

## Abstract

Synthetic macrocycles have served as principal tools for supramolecular chemistry since their establishment, and the investigation of macrocycles‐aided organic‐inorganic hybrid nanomaterials has also attracted broad interest in chemistry and material communities during the past decade owing to their widespread applications in optical sensing, catalytic degradation, biomedicine, and other related fields. Herein, a new class of silver nanoparticles (AgNPs) modified by anionic water‐soluble [2]biphenyl‐extended pillar[6]arene (WBpP6), namely WBpP6‐AgNPs, is designed and synthesized through a facile one‐pot method. WBpP6‐AgNPs with good dispersion and stability exhibit efficient catalytic properties toward the hydrogenation of a series of aromatic nitro compounds and also show good performance in label‐free detection toward diquat.

## Graphical abstract

In this work, a new type of macrocycle‐modified silver nanoparticles, namely WBpP6‐AgNPs, is successfully designed and prepared through a facile one‐pot strategy, which not only exhibits efficient catalytic properties toward the hydrogenation of a series of aromatic nitro compounds but also shows good performance in label‐free detection toward diquat.

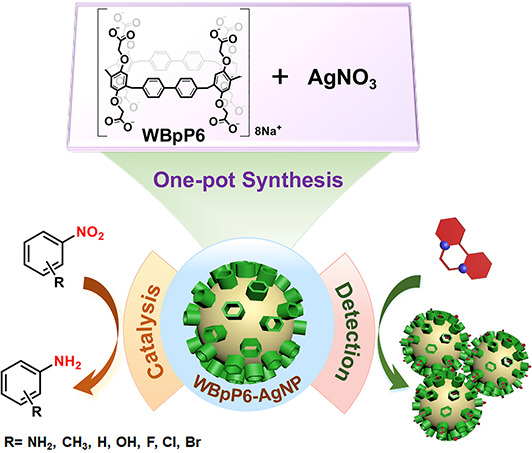

## 1 INTRODUCTION

With molecular recognition and complexation properties, macrocyclic compounds are the fundamental workhorses in supramolecular chemistry and materials science.^[1]^ Due to their synthetic accessibility, ease of functionalization, preorganized cavity structures, and predictable non‐covalent interactions, macrocyclic chemistry has created a boom in many research fields during the past decades, giving rise to practical or potential applications in, for example, stimuli‐responsive supramolecular systems,[^2^, ^3^, ^4^] biosensing and imaging,[^5^, ^6^] catalysis,[^7^, ^8^] and many others. Inspired by pillararenes,[^9^, ^10^, ^11^] a new type of synthetic macrocyclic arene, namely [2]biphenyl‐extended pillar[6]arene ([2]Bp‐ExP6), was first reported by us in 2016,^[12]^ and its derivatization and supramolecular functions have been well established during the past five years.[^13^, ^14^, ^15^] For instance, we designed and synthesized a series of [2]Bp‐ExP6 derivatives in 2018, in which the cationic water‐soluble version exhibited good binding affinity to naphthalenesulfonate substrates,^[13]^ and the anionic water‐soluble version was used to construct functional host‐guest systems in solution for controlled drug delivery.^[15]^ These pioneering studies have laid the groundwork for extending [2]Bp‐ExP6 and its derivatives to other research fields.

Metal nanoparticles have attracted much attention in chemistry and material communities during the past decades,[^16^, ^17^, ^18^, ^19^, ^20^, ^21^, ^22^, ^23^] among which silver nanoparticles (AgNPs) with unique stability, uniformity, and biocompatibility exhibited widespread applications in many research fields, including but not limited to optical sensing,[^24^, ^25^, ^26^, ^27^] catalytic degradation,[^28^, ^29^, ^30^, ^31^] biomedicine,[^32^, ^33^, ^34^] electronics[^35^, ^36^, ^37^] and antibacterial.[^38^, ^39^, ^40^] However, on account of the high surface energy and uncontrollable aggregation and precipitation, the development of AgNPs has been restricted to a great extent.[^41^, ^42^] One of the effective methods to address this issue is to modify AgNPs with macrocyclic aromatic skeleton to prevent the undesirable overgrowth and aggregation of AgNPs.[^43^, ^44^, ^45^, ^46^] For example, Zhao et al. reported a carboxylate pillar[6]arene‐modified AgNPs‐functionalized 2D hybrid material (WP6@Ag@COF) in 2019, displaying enhanced sensing capability for paraquat.^[43]^ Diao and coworkers reported a hybrid nanomaterial (CP5‐AgNPs/GO) for the electrochemical detection of paraquat, where CP5 was used as a capping ligand on AgNPs' surface.^[44]^ Inspired by these pioneering works and in conjunction with our continuous efforts on organic‐inorganic hybrid nanoplatforms, we conjecture that a new type of hybrid AgNPs with both host‐guest binding ability and optical property/response can be constructed by capping them with [2]Bp‐ExP6.

**FIGURE 1:**
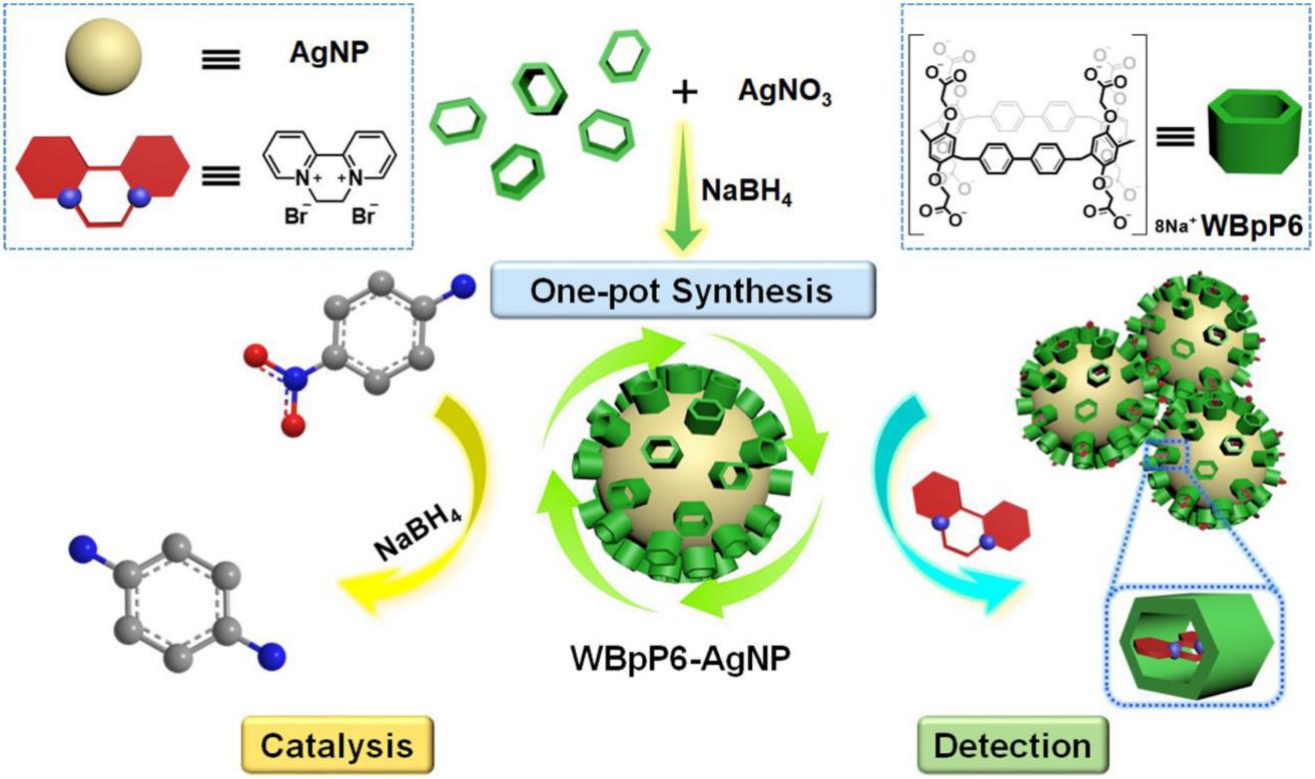
Schematic illustration of the one‐pot synthesis of WBpP6‐AgNPs for efficient catalysis and sensitive detection.

Herein, we report a new class of AgNPs modified by anionic water‐soluble [2]Bp‐ExP6, namely WBpP6‐AgNPs, which combines the merits of good dispersion and stability. This material exhibits excellent catalytic properties toward hydrogenating a series of aromatic nitro compounds by accelerating the electron transfer process. Interestingly, WBpP6‐AgNPs show good performance of label‐free detection of diquat owing to the host‐guest properties of WBpP6 (Figure 1).

## 2 RESULTS AND DISCUSSION

WBpP6 was synthesized based on our previously published procedure (Scheme S1, Figures S1‐S6).^[15]^ Starting from the permethylated [2]Bp‐ExP6 (MeBpP6), demethylation, etherification, hydrolysis, acidification, and deprotonation were carried out successively to obtain the target product. Furthermore, the noncyclic monomeric analog (M) was synthesized as the controlled group by the same synthesis method (Scheme S2, Scheme S3, Figures S7‐S15). WBpP6‐ or M‐modified AgNPs were constructed by reducing AgNO_3_ with NaBH_4_ in the presence of WBpP6 or M, respectively.

**FIGURE 2:**
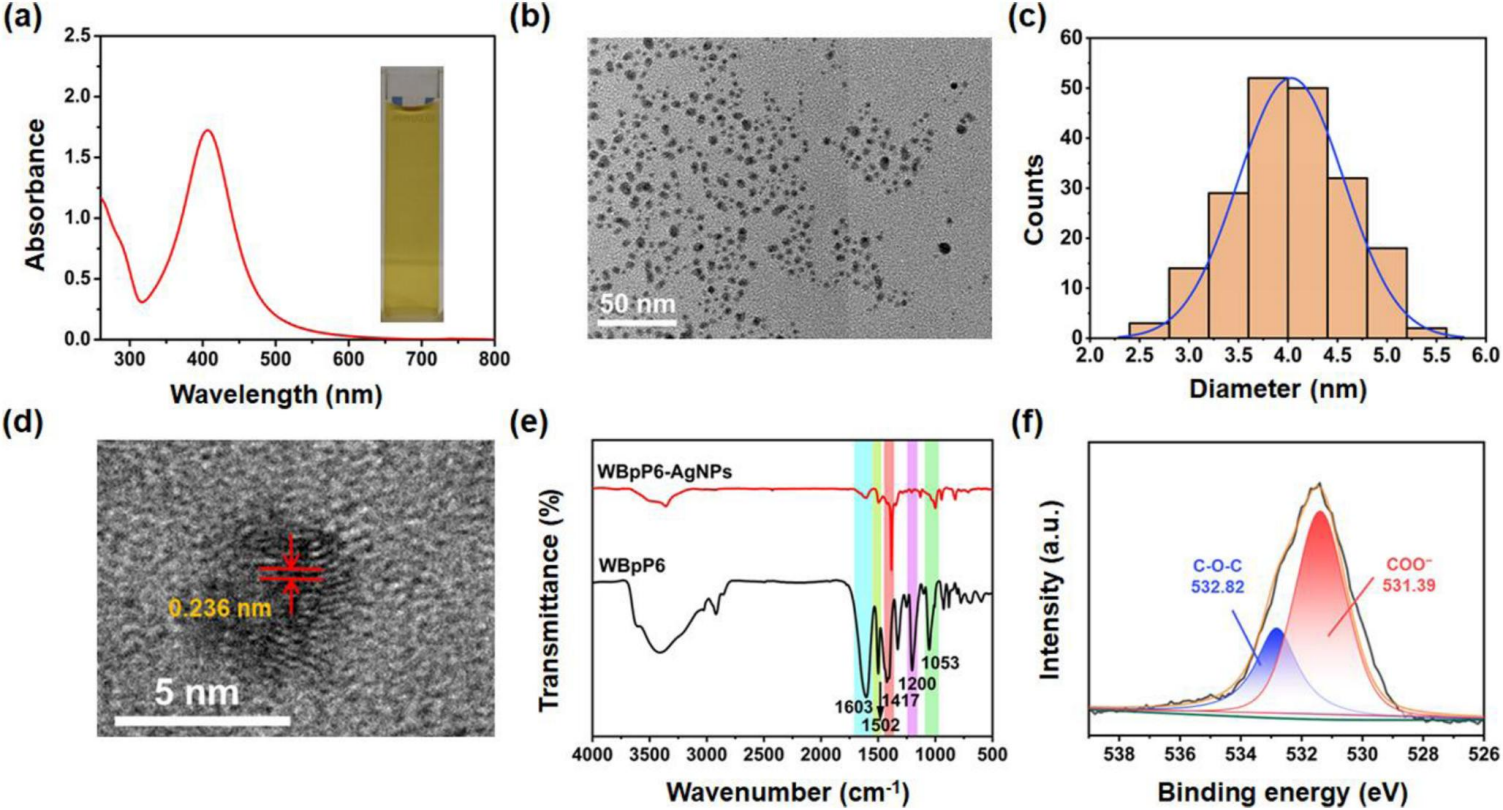
(a) UV‐vis spectrum of WBpP6‐AgNPs solution. Inset: photograph of WBpP6‐AgNPs solution. (b) TEM image of WBpP6‐AgNPs. (c) The histogram of WBpP6‐AgNPs size distribution based on TEM image (based on 200 particles). (d) The HR‐TEM image of WBpP6‐AgNPs. (e) FT‐IR spectra of WBpP6 and WBpP6‐AgNPs. (f) XPS spectrum (O 1s) of WBpP6‐AgNPs.

WBpP6‐AgNPs and M‐AgNPs were fully characterized by transmission electron microscopy (TEM), UV‐vis spectroscopy, X‐ray photoelectron spectroscopy (XPS), dynamic light scattering (DLS), Fourier transform infrared (FT‐IR) spectroscopy and zeta‐potential (Figure 2; Figures S17 and S18). The surface plasmon resonance (SPR) of AgNPs observed at 405 nm through the UV‐vis spectrum of WBpP6‐AgNPs colloids revealed the successful preparation of uniform WBpP6‐AgNPs (Figure 2a).[^47^, ^48^] Meanwhile, WBpP6‐AgNPs exhibited a spherical morphology with high dispersity in the colloidal solution, as revealed by the TEM image (Figure 2b). The average diameter of WBpP6‐AgNPs calculated using statistical analysis was determined to be 4.0 ± 1.3 nm, which was smaller and more uniform than that of M‐AgNPs (2.8–12.3 nm) (Figure 2c and Figure S18). The high‐resolution TEM image indicated that WBpP6‐AgNPs possessed well‐defined crystalline planes with lattice fringes of 0.236 nm (Figure 2d), in accordance with the (111) plane, for face‐centered‐cubic (fcc) Ag. As shown in the FT‐IR spectra of WBpP6‐AgNPs (Figure 2e), the characteristic peaks at ∼1603 and ∼1502 cm^−1^ were ascribed to the C=C stretching mode of the benzene ring within WBpP6. The peak located at ∼1417 cm^−1^ arose from the −COO^−^ stretching vibration and the peaks at ∼1200 and ∼1053 cm^−1^ could be attributed to the Ar−O−C group. Furthermore, the XPS spectrum was further used to test the interactions between WBpP6 and AgNPs. For the O 1s XPS spectrum of WBpP6 (Figure S19), the peaks at 532.82 and 531.26 eV could be assigned to ether oxygen atoms and the two comparable oxygen atoms of the carboxylate (−COO^−^) moiety. Additionally, in the O 1s spectrum of WBpP6‐AgNPs, the peak of the carboxylate oxygen atoms was shifted to 531.39 eV due to the O−Ag coordination bond along with the peak of Sodium Auger (Na KLL) disappearing (Figure 2f).^[49]^ All these results confirmed the successful modification of WBpP6 on the surface of AgNPs. In addition, on account of the more negative zeta potential of WBpP6‐AgNPs than M‐AgNPs (Figures S17b and S18d), WBpP6‐AgNPs possessed better stability and could be stored for several months without any changes in color and SPR spectrum (Figure S20).

As mentioned above, WBpP6 was crucial for the synthesis of AgNPs for the following two aspects: on the one hand, WBpP6 was modified on the surface of AgNPs through O−Ag coordination bonds, which limited the growth of AgNPs. On the other hand, the skeletons of WBpP6 and the electrostatic repulsion of carboxylic groups prevented the aggregation of AgNPs and maintained the system's stability.

**FIGURE 3:**
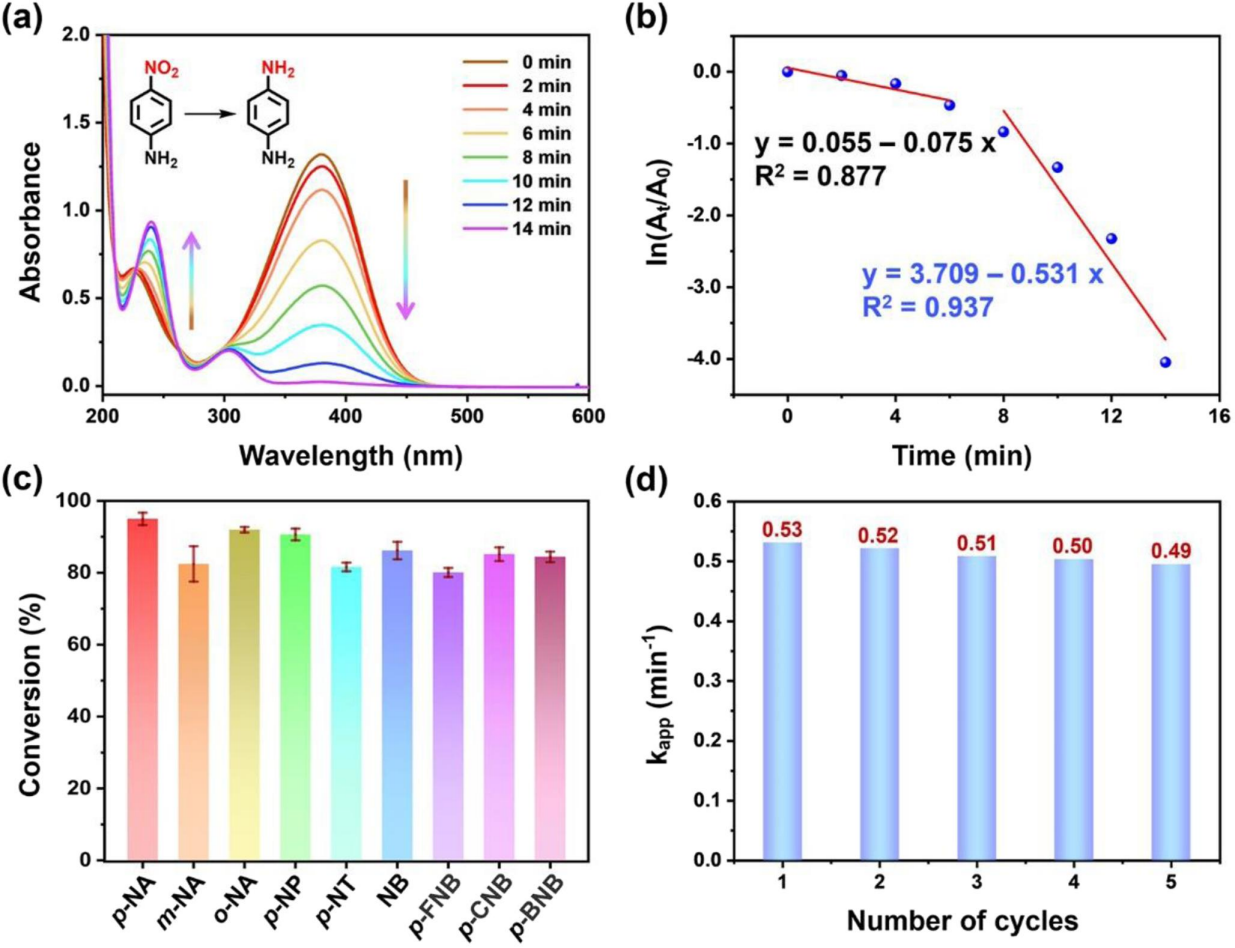
(a) UV‐vis spectra of *p*‐NA solution reduced by NaBH_4_ using WBpP6‐AgNPs as a catalyst. (b) The ln (*A*_*t*_/*A*_*0*_) versus time for the catalytic reduction of *p*‐NA. (c) The conversion efficiencies for the reductions of aromatic nitro compounds using WBpP6‐AgNPs as a catalyst. (d) Stability of the WBpP6‐AgNPs catalyst reduction of *p*‐NA over five cycles at 298 K. *p*‐NA, *m*‐nitroaniline (*m*‐NA), *o*‐nitroaniline (*o*‐NA), *p*‐nitrophenol (*p*‐NP), *p*‐nitrotoluene (*p*‐NT), nitrobenzene (NB), *p*‐fluoronitrobenzene (*p*‐FNB), *p*‐chloronitrobenzene (*p*‐CNB) and *p*‐bromonitrobenzene (*p*‐BNB).

Since organic catalysis is one of the significant applications of AgNPs, we explored the catalytic activity of WBpP6‐AgNPs toward reducing a set of aromatic nitro compounds to the corresponding amino compounds. At the outset, *p*‐nitroaniline (*p*‐NA) was selected as the substrate, and the reaction was carried out in the presence of different amounts of WBpP6‐AgNPs (Figure 3a and Figure S21). As in Figure S21a in the Supporting Information, the absorption peak at 380 nm remains unchanged for more than 6 hours in the absence of WBpP6‐AgNPs, demonstrating that *p*‐NA can hardly be reduced by NaBH_4_ itself. However, with the addition of WBpP6‐AgNPs, the absorption peak at 380 nm gradually decreased, accompanied by the emergence of new peaks at 240 and 300 nm, which could be attributed to the target reduction product *p*‐phenylenediamine (*p*‐PDA), indicating the reduction reaction proceeded smoothly. Moreover, the fading process of the reaction system could also be visually monitored (from yellow to colorless). Subsequently, with the increase in WBpP6‐AgNPs content, the reaction rate rose gradually. Until the amount of WBpP6‐AgNPs reached 50 μL (86.7 μg/mL), the continuous addition of WBpP6‐AgNPs had little influence on the reaction rate. Therefore, we speculated that the increase in the content of WBpP6‐AgNPs enhanced the probability of reactant molecules adsorbed on the surface of nanoparticles and accelerated the electron transfer process from BH_4_^−^ to *p*‐NA, thus accelerating the reduction reaction.

With optimal catalyst loading (50 μL, 86.7 μg/mL), we then explored the scope of the reaction on other aromatic nitro compounds. The position and electronic effects of substituents were investigated, including *p*‐NA, *m*‐nitroaniline (*m*‐NA), *o*‐nitroaniline (*o*‐NA) and a series of nitrobenzene compounds with electron‐withdrawing or electron‐donating groups (such as alkyl groups, H, OH, F, Cl and Br) (Figures S22‐S25). The linear correlation between ln (*A*_*t*_/*A*_*0*_) and reaction time (*t*) demonstrated that the reductions of aromatic nitro compounds conformed to the pseudo‐first‐order kinetics in the presence of excessive NaBH_4_ in the reaction system.^[50]^ The catalytic performances of WBpP6‐AgNPs were quantitatively evaluated using the conversion efficiencies and the apparent rate constant *k*_*app*_ based on the formula of ln(*A*_*t*_/*A*_0_) = ln(*C*_*t*_/*C*_0_) = −*k*_*app*_ *t* (Figure 3b,c).^[51]^ The experimental results showed that all the reactants could be effectively reduced to the corresponding amine products with over 80% conversions (Figure 3c). Moreover, as the controlled group, the catalytic activity of M‐AgNPs, which were not coated with the nanoporous layer of WBpP6 rings on AgNPs toward reducing *p*‐NA, was tested under identical conditions (Figures S26‐S27). The *k*_*app*_ value of 0.243 min^−1^ was lower than that of WBpP6‐AgNPs (0.531 min^−1^) due to the larger and unevener particle size of M‐AgNPs.

Moreover, the graphs of ln(*A*_*t*_/*A*_*0*_) versus time demonstrated that all the catalytic reductions of aromatic nitro compounds could be divided into two stages. At the initial stage, part of substrate preferentially contacted the bare surface of AgNPs. Then, the other part of the substrate could also contact with AgNPs through the cavity of WBpP6 based on the host‐guest interactions. The larger substrate‐accessible surface facilitated the electron transferred from BH_4_^−^ to substrate molecule, so the catalytic reduction process could be accelerated.[^48^, ^52^] More importantly, WBpP6‐AgNPs could be recycled at least five times without any obvious reduction in the catalytic performance, indicating the excellent recyclability and stability of WBpP6‐AgNPs (Figure 3d).

In comparison with traditional metal nanoparticles, the catalysis based on WBpP6‐AgNPs possessed two major advantages: on the one hand, the nanoporous layer of carboxylate‐macrocycles adsorbed on the surfaces of AgNPs endows them with remarkable stability and enhances the homogeneous morphology of WBpP6‐AgNPs, thereby improving the catalytic activity. On the other hand, the nanoporous layer of WBpP6 could potentially capture the aromatic nitro compounds through the host‐guest recognition interactions, facilitating contact with the AgNPs surface.

**FIGURE 4:**
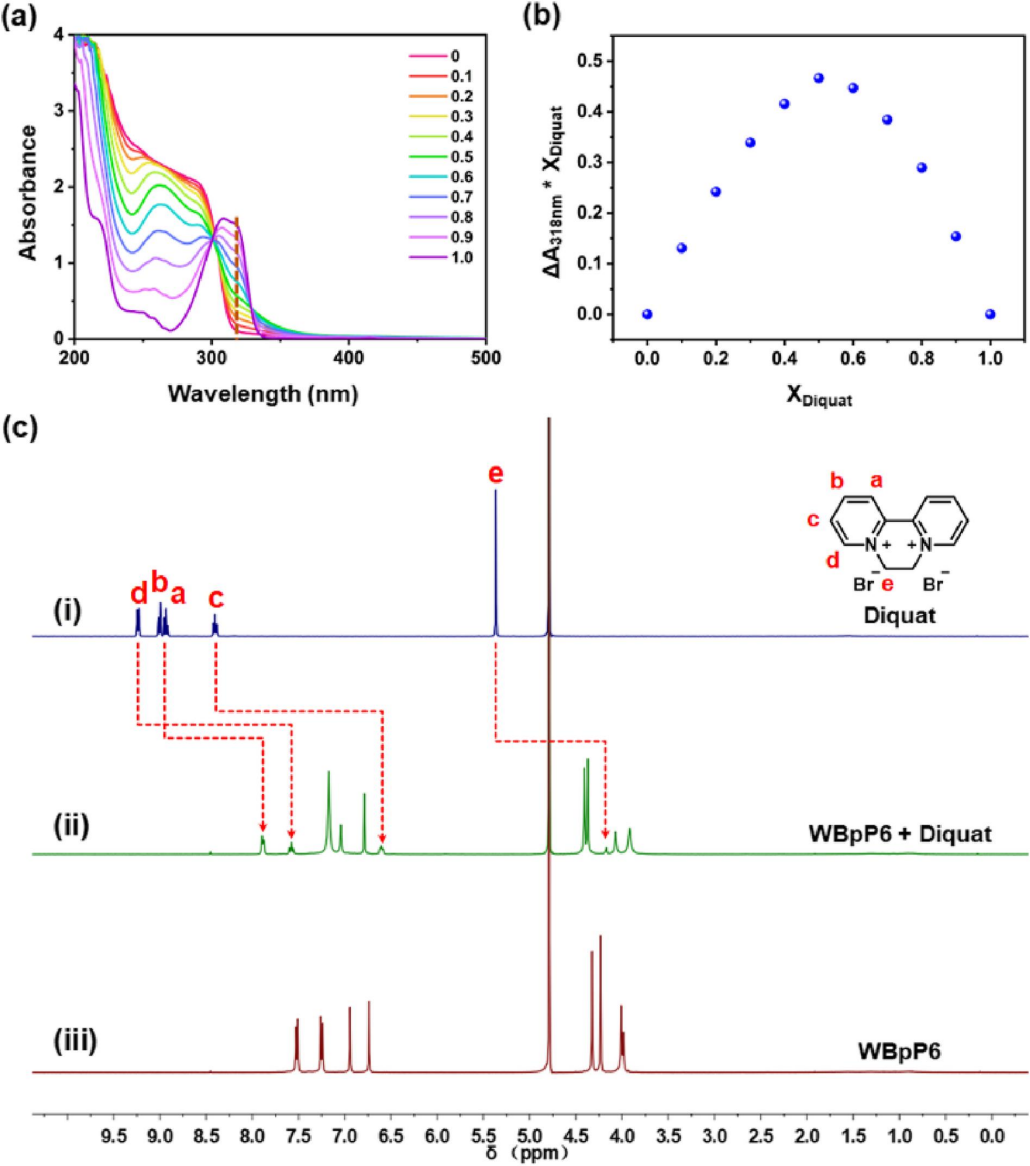
(a) UV‐vis spectra of different mole fractions of diquat and WBpP6 solution. (b) Job's plot obtained by recording the absorbance at 318 nm for the solution of diquat and WBpP6 at 298 K. (c) ^1^H NMR spectra (400 MHz, D_2_O, 298 K) of (i) diquat, (ii) WBpP6 and diquat (1:1 molar ratio), (iii) WBpP6 at 5.0 mM.

Because of the host‐guest recognition properties of WBpP6, we envision that WBpP6‐AgNPs have potential application as a simple and feasible optical probe. Diquat, one of the most widely used toxic herbicides, possessed a suitable molecular size matching the cavity size of WBpP6 (Scheme S3 and Figure S16). Therefore, diquat was chosen as the model cationic guest. The host‐guest binding capability between WBpP6 and diquat was first investigated by UV‐vis spectroscopy and ^1^H NMR spectra of a 1:1 host‐guest mixture in water (Figure 4 and Figure S27). As shown in the UV‐vis spectra in Figure 4a, the absorbance of the solution of diquat and WBpP6 at 318 nm increased along with the increase in the mole fraction of diquat. And the Job's plot obtained by recording the absorbance at 318 nm for the solution of diquat and WBpP6 at 298 K indicates the 1:1 stoichiometry of diquat and WBpP6 (Figure 4b). Moreover, compared with the free guest, all protons of diquat shifted upfield upon the addition of WBpP6 as a result of the inclusion‐induced shielding effect (Δ*δ* = −1.06, −1.10, −1.80, −1.66, −1.20 ppm for protons a, b, c, d and e, respectively) (Figure 4c). These observations indicated that diquat was fully threaded into the cavity of WBpP6, thus forming a corresponding stable host‐guest inclusion complex.

**FIGURE 5:**
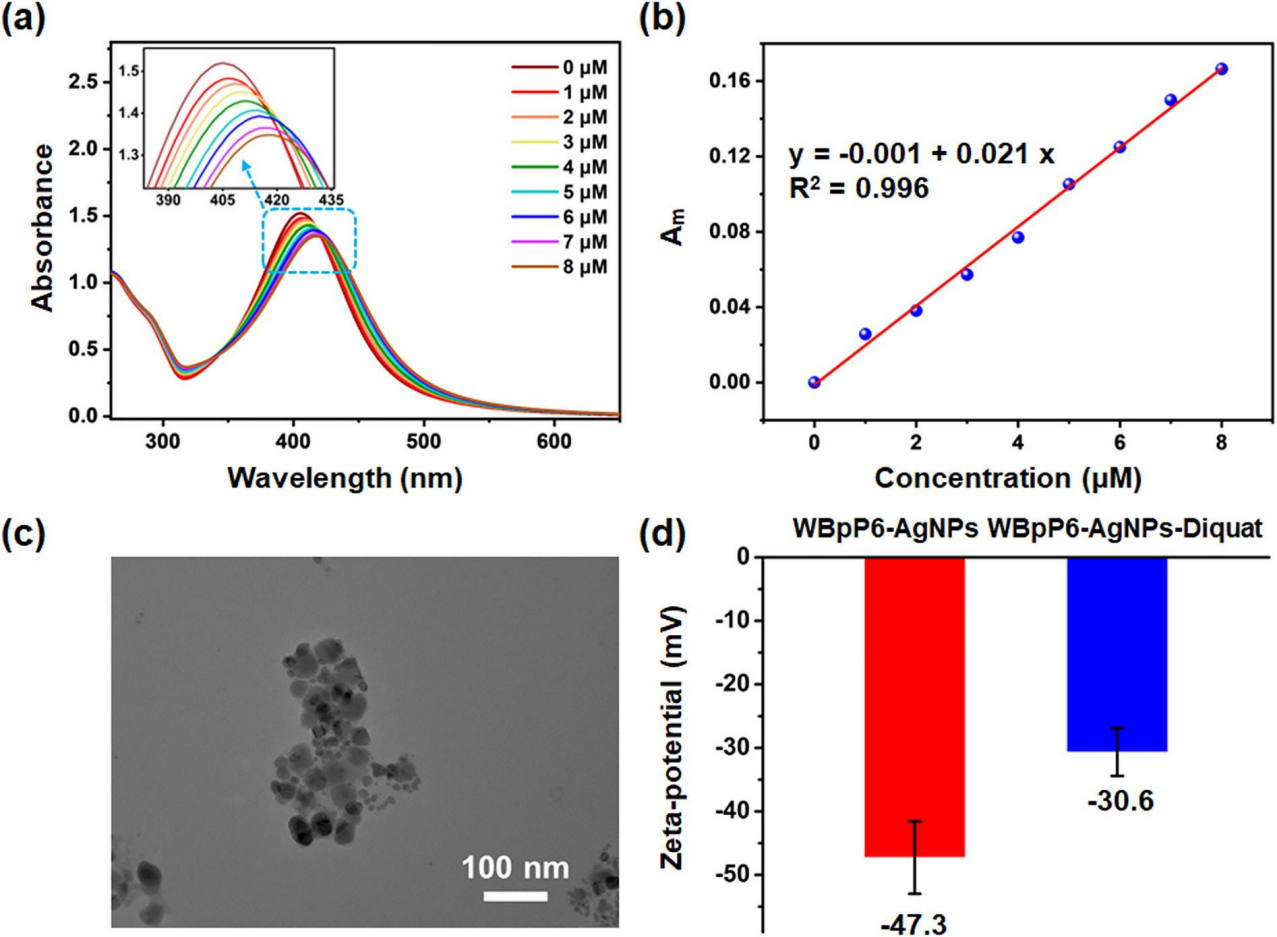
(a) UV‐vis spectra of WBpP6‐AgNPs mixed with different concentrations of diquat. (b) Relationship between decreasing absorbance of WBpP6‐AgNPs at 405 nm (*A*_*m*_) and the diquat concentration ranging from 0 to 8.0 μM. (c) TEM image of WBpP6‐AgNPs with diquat (100.0 μM). (d) Zeta‐potential values of the WBpP6‐AgNPs and WBpP6‐AgNPs‐Diquat (100.0 μM).

As shown in Figure 5a, along with the addition of different concentrations of diquat, the SPR peak of WBpP6‐AgNPs gradually underwent a bathochromic shift and a decrease in absorbance, which could be attributed to the aggregation of WBpP6‐AgNPs driven by the synergy of host‐guest complex and the destruction of the original electrostatic balance of WBpP6 and diquat. Furthermore, an excellent linear correlation between the decreasing absorbance of WBpP6‐AgNPs at 405 nm (*A*_*m*_) and the concentrations of diquat (0–8.0 μM) was observed (Figure 5b), in which *A*_*m*_ stands for the value calculated by the equation [*A*_405_(0)‐*A*_405_(m)]/*A*_405_(0), *A*_*405*_(0) stands for the absorbance of WBpP6‐AgNPs in the absence of diquat and *A*_*405*_(m) represents the absorbance of WBpP6‐AgNPs with different concentrations of diquat at 405 nm. Moreover, the TEM image showed the aggregation of WBpP6‐AgNPs‐Diquat (Figure 5c), and the decrease in the zeta‐potential value of WBpP6‐AgNPs‐Diquat shown in Figure 5d further confirmed the occurrence of aggregation. Additionally, the controlled experiment was first performed by replacing the diquat solution with equal H_2_O (Figure S28a). The result showed that there was no change in *A*_*m*_, illustrating that the changes in *A*_*m*_ were not caused by pure water. Subsequently, a controlled experiment was also carried out to illustrate the synergistic effect of WBpP6‐AgNPs (Figure S28b). Furthermore, the limit of detection (LOD) for diquat was calculated to be 6.5 × 10^−8^ M. All the results suggested above that WBpP6‐AgNPs, a kind of new hybrid material, could be utilized as nano‐sensors toward qualitatively detecting the herbicide diquat, which may have great prospects in the field of chemical detection applications.

## 3 CONCLUSION

In summary, we have presented the synthesis of new silver nanoparticles modified with anionic water‐soluble extended pillar[6]arene, that is, WBpP6‐AgNPs. WBpP6‐AgNPs were prepared through a facile one‐pot method in an aqueous solution with good dispersibility and stability. More importantly, the resulting organic‐inorganic hybrid WBpP6‐AgNPs showed excellent catalytic activity to reduce a series of aromatic nitro compounds, and the efficiency of conversions was over 80%. Meanwhile, WBpP6‐AgNPs could be recycled at least five times. By the host‐guest interactions between WBpP6 and diquat, the performance of label‐free detection of WBpP6‐AgNPs toward diquat was studied by UV‐vis spectroscopy in the diquat concentration range of 0–8.0 μM with LOD of 6.5 × 10^−8^ M. Overall, the marriage of water‐soluble supramolecular macrocycle WBpP6 and AgNPs offers a valuable strategy for fabricating organic‐inorganic hybrid nanomaterials, which hold great potential in sensing and catalysis applications.

## CONFLICT OF INTEREST STATEMENT

The authors declare no conflicts of interest.

## ETHICS STATEMENT

No animal or human experiments were involved in this study.

## Supporting information

Supporting Information S1

## Data Availability

The data that support the findings of this study are available in the supplementary material of this article.
