# Supplementary material for: [2]Biphenyl‐extended pillar[6]arene functionalized silver nanoparticles for catalysis and label‐free detection
Source: Smart Mol. 2023 Nov 14;1(3):e20230016. doi: 10.1002/smo.20230016 (PMC12118214; doi:10.1002/smo.20230016)
Supplement: Supplementary file 1 — Supporting Information S1 [file SMO2-1-e20230016-s001.docx]

Supporting Information

**[2]Biphenyl-Extended Pillar[6]arene Functionalized Silver Nanoparticles for Catalysis and Label-Free Detection**

*Dongxia Li, Gengxin Wu, Xin Wang, Jia-Rui Wu,* and Ying-Wei Yang**

D. Li, G. Wu, Dr. X. Wang, Prof. J.-R. Wu, Prof. Y.-W. Yang

International Joint Research Laboratory of Nano-Micro Architecture Chemistry, College of

Chemistry, Jilin University, Changchun 130012, P. R. China

E-mail: jrwu@jlu.edu.cn (J.-R.W.); ywyang@jlu.edu.cn (Y.-W.Y.)

Prof. J.-R. Wu

Key Laboratory of Automobile Materials of Ministry of Education and School of Materials

Science and Engineering, Jilin University, Changchun 130025, P. R. China

**Table of contents**

1. Materials and methods ············································································· S2

2. Synthesis methods·················································································· S2

3. Catalysis method ················································································· S14

4. Detection method················································································· S14

5. Supplementary data ·············································································· S15

6. References························································································· S23

**1. Materials and methods**

**Materials**: All the chemicals and solvents were purchased from commercial sources and used as received unless otherwise noted. Ultrapure water, purified by Experimental Water System (Lab-UV-20), was used in relevant experiments.

**Methods**: 1H and 13C NMR spectra were recorded on a Bruker AVANCE III-400 MHz

instrument at 298 K and a Bruker Avance DMX 300 instrument at room temperature. HRMS were obtained on an Agilent1290-Bruker micrOTOF QII. Ultraviolet-visible (UV-vis) spectra were collected on a Japan Shimadzu UV-1900 instrument. Fourier transform infrared (FT-IR) spectra were recorded on a Vertex 80 V spectrometer. The XPS spectra were obtained with a Shimadzu/Krayos AXIS Ultra DLD. Transmission electron microscopy (TEM) images were obtained on a FEI TECNAI F20 at an accelerating voltage of 200 kV. The zeta-potential value and hydrodynamic diameters of dynamic light scattering (DLS) were measured on an OMEC NS-90Z Nanoparticle size and zeta potential analyzer.

**2. Synthesis methods**

**2.1 Synthesis of WBpP6**S1


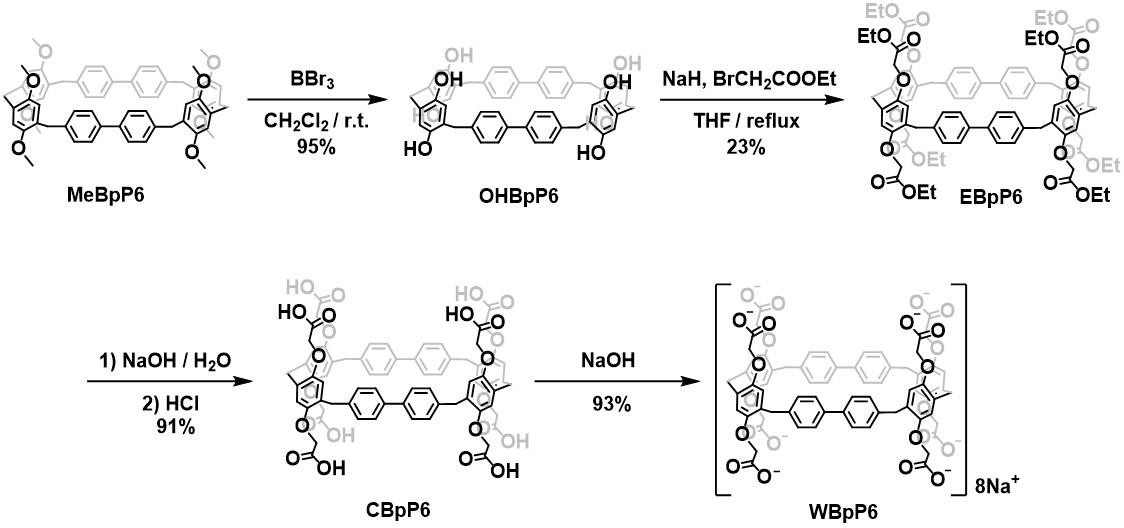


**Scheme S1.** Synthetic route to WBpP6.


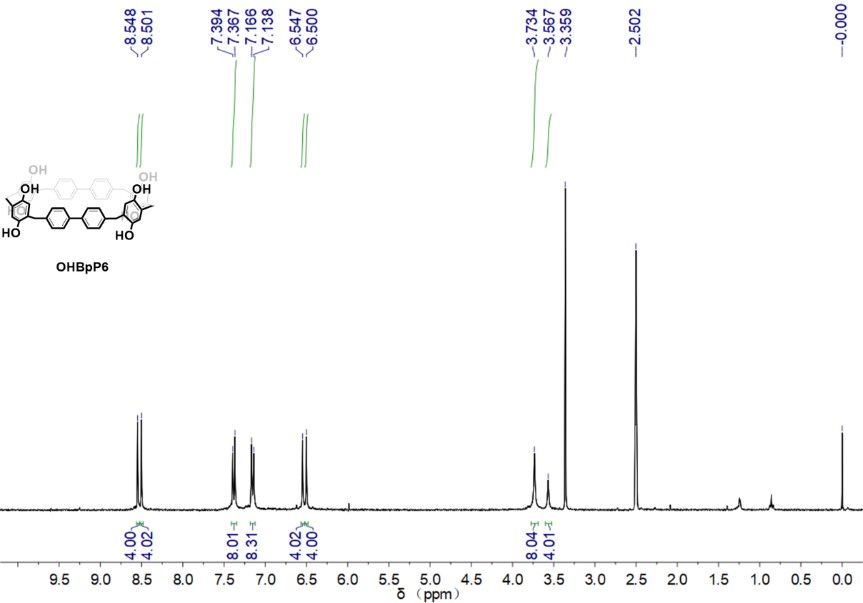


**Figure S1.** 1H NMR spectrum (300 MHz, DMSO-*d6*, 298 K) of OHBpP6.


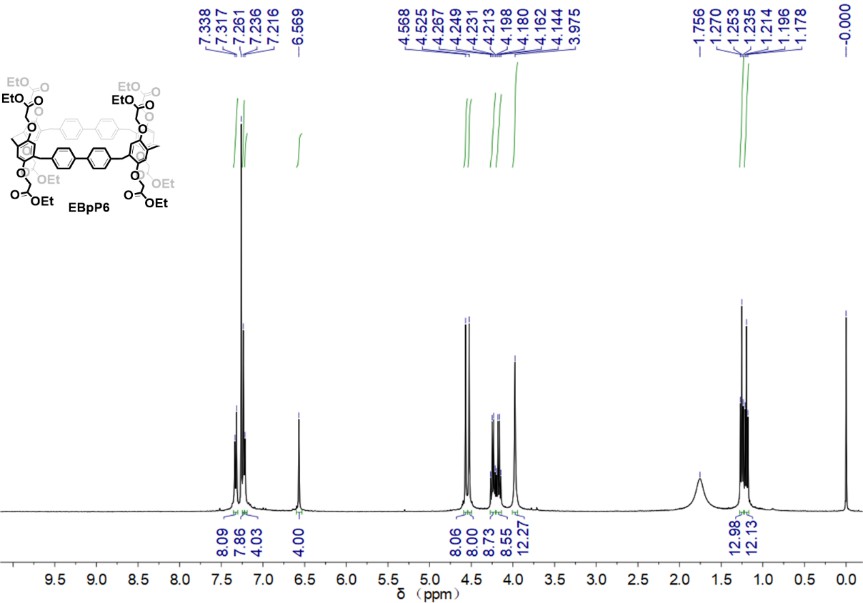


**Figure S2.** 1H NMR spectrum (300 MHz, CDCl3, 298 K) of EBpP6.


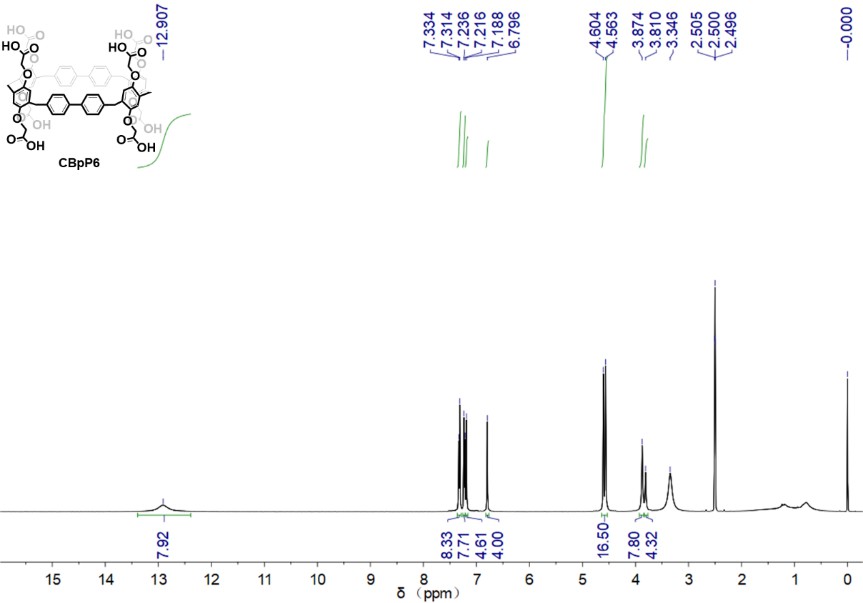


**Figure S3.** 1H NMR spectrum (400 MHz, DMSO-*d6*, 298 K) of CBpP6.


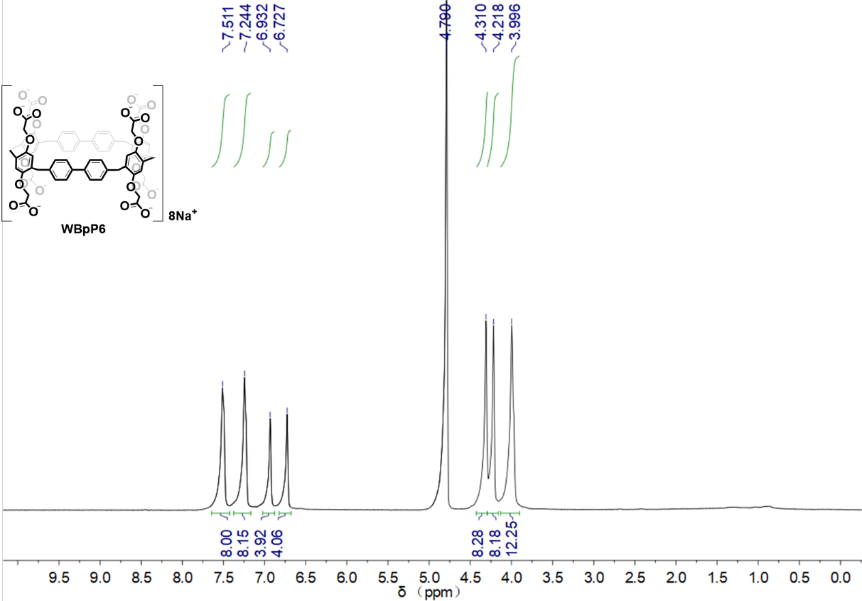


**Figure S4.** 1H NMR spectrum (400 MHz, D2O, 298 K) of WBpP6.


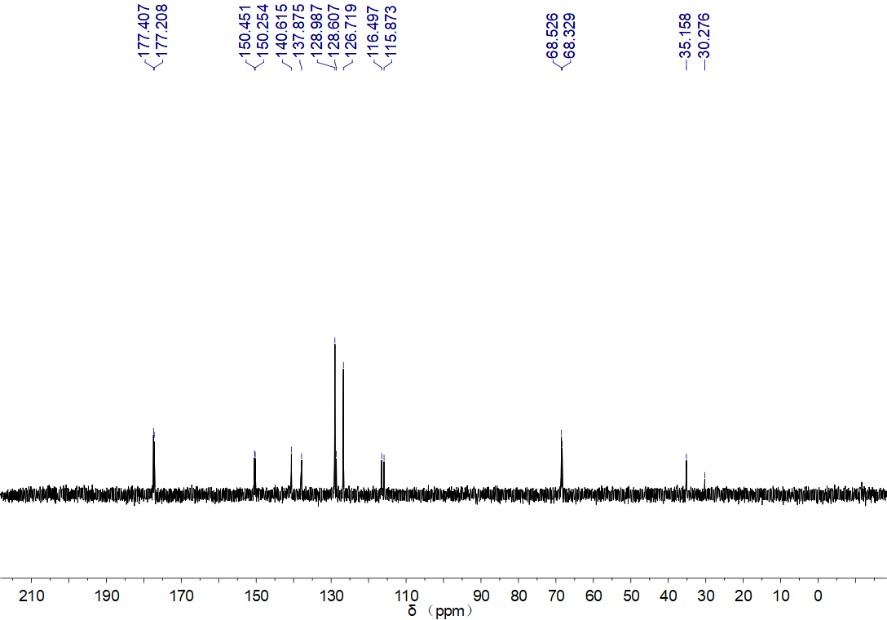


**Figure S5.** 13C NMR spectrum (101 MHz, D2O, 298 K) of WBpP6.

Intens.

6000

2- -MS, 3.2min #191

1284.3693

5000

4000

3000

2-

641.1695

2000

1000

956.2641


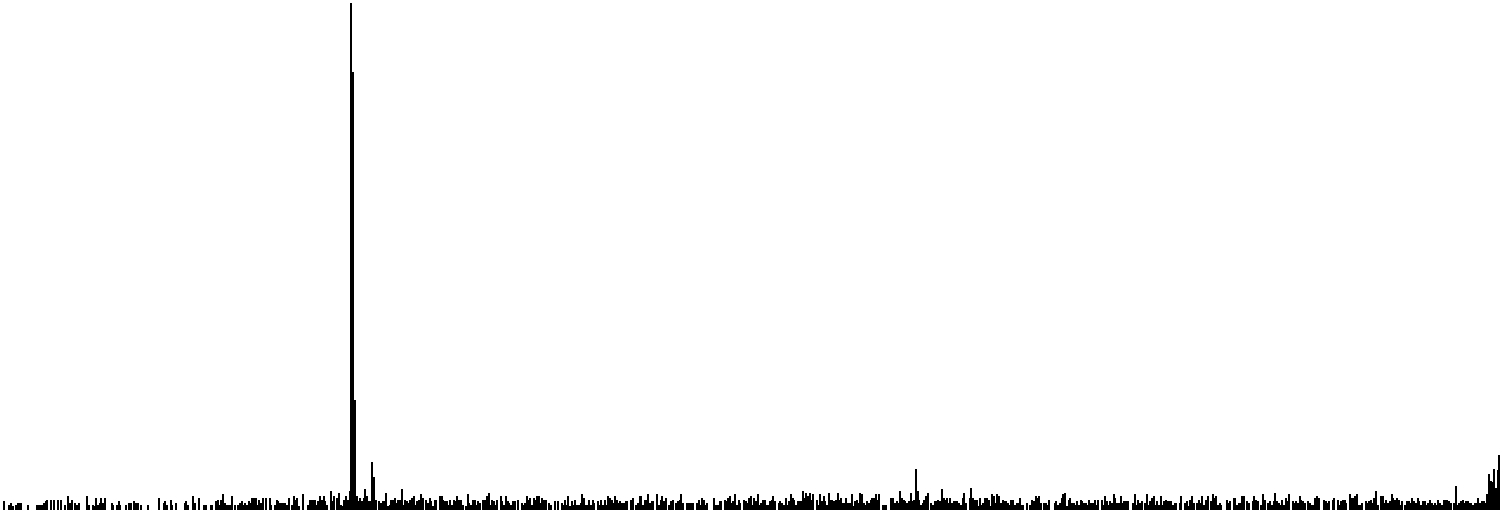

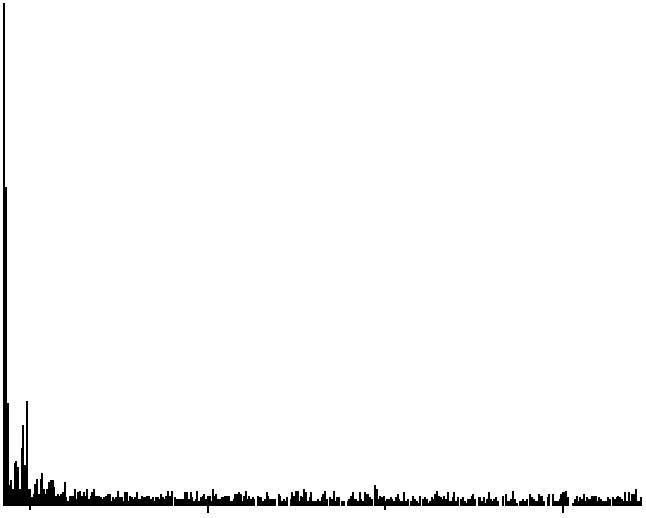


0

600 800 1000 1200 1400 1600 m/z

**Figure S6.** HRMS spectrum of WBpP6: [M-8Na++6H+]2- calcd for C70H58O242-, 641.1653, found 641.1695; [M-8Na++8H+] calcd for C70H60O24, 1284.3475, found 1284.3693.

**2.2 Synthesis of M**S2


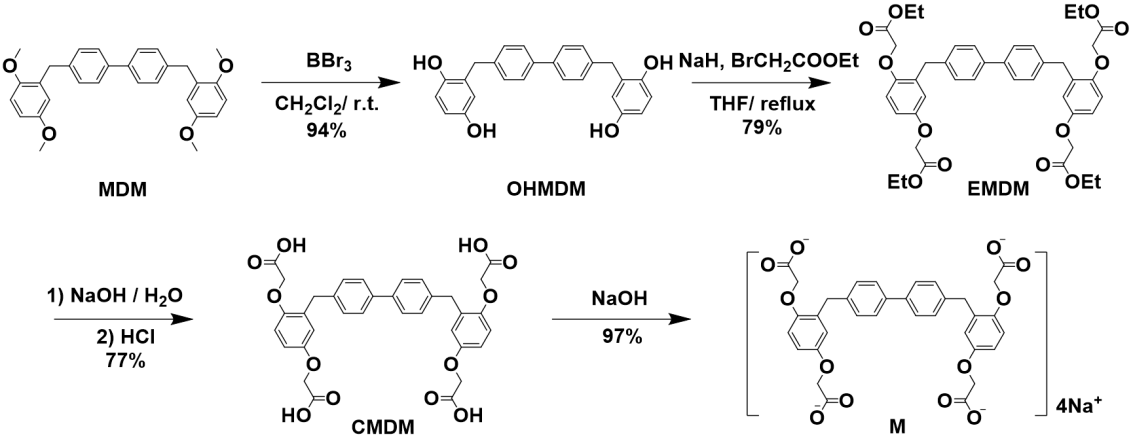


**Scheme S2.** Synthetic route to M.

**Synthesis of OHMDM**: Dry CH2Cl2 (60 mL) was added into a flask. Then MDM (3.18 g, 7.0 mmol) was added tardily. After ice bathing for 10 min, boron tribromide (8.05 mL, 84.0 mmol) was added into the reaction flask and stirred at 25 °C for 3 d under a nitrogen atmosphere. Then the mixture was quenched with water. After stirring the reaction mixture for another 3 d, OHMDM (2.62 g, 94%) was obtained as a white solid by filtration and recrystallization from acetone and water. 1H NMR (400 MHz, DMSO-*d6*, 298 K): δ 8.67 (s, 2H), 8.55 (s, 2H), 7.51 (d, *J* = 8.1 Hz, 4H), 7.26 (d, *J* = 8.1 Hz, 4H), 6.61 (d, *J* = 8.4 Hz, 2H), 6.44 (dd, *J* = 10.7, 5.5

Hz, 4H), 3.81 (s, 4H).13C NMR (101 MHz, DMSO-*d6*, 298 K): δ 150.22, 147.81, 140.79, 138.10,

129.71, 128.43, 126.80, 117.27, 116.11, 113.87, 35.40.


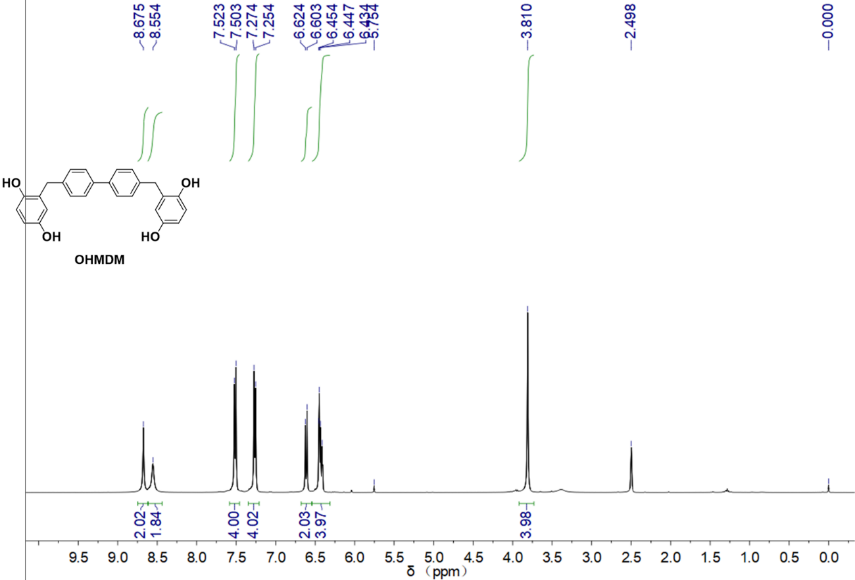


**Figure S7.** 1H NMR spectrum (400 MHz, DMSO-*d6*, 298 K) of OHMDM.


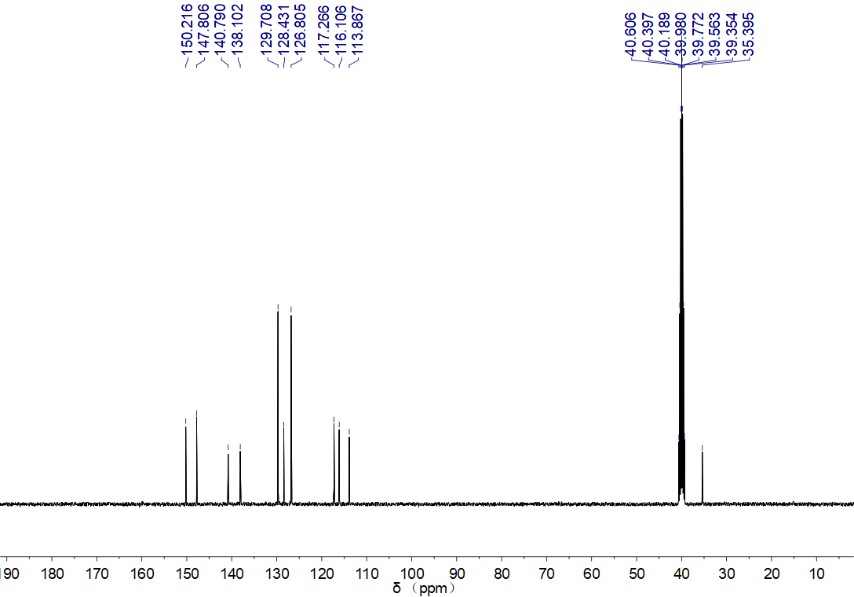


**Figure S8.** 13C NMR spectrum (101 MHz, DMSO-*d6*, 298 K) of OHMDM.

**Synthesis of EMDM**: OHMDM (1.39 g, 3.5 mmol) and NaH (0.67 g, 28.0 mmol) were dissolved in THF (50 mL). And ethyl bromoacetate (3.1 mL, 28.0 mmol) was added at room temperature. Then the reaction mixture was heated at 65°C in an oil bath for 72 h. After cooling to room temperature, the mixture was quenched with water. The organic layer was collected and concentrated, and EMDM (2 g, 79%) was collected by column chromatography (dichloromethane/ethyl acetate= 50/1, v/v). 1H NMR (400 MHz, CDCl3, 298 K): δ 7.46 (d, *J* =

8.2 Hz, 4H), 7.29 (d, *J* = 8.2 Hz, 4H), 6.76 (d, *J* = 2.3 Hz, 2H), 6.69 (d, *J* = 2.8 Hz, 4H), 4.56 (s, 4H), 4.52 (s, 4H), 4.26 (dd, *J* = 9.3, 4.9 Hz, 5H), 4.22 (dd, *J* = 9.3, 4.9 Hz, 5H), 4.04 (s, 4H),

1.29 (t, *J* = 6.7 Hz, 7H), 1.25 (t, *J* = 6.8 Hz, 7H). 13C NMR (101 MHz, CDCl3, 298 K): δ 169.14,

169.10, 152.62, 150.78, 139.31, 138.81, 132.00, 129.44, 126.93, 117.93, 112.93, 112.56, 66.58,

66.12, 61.30, 61.24, 35.68, 14.20, 14.17.


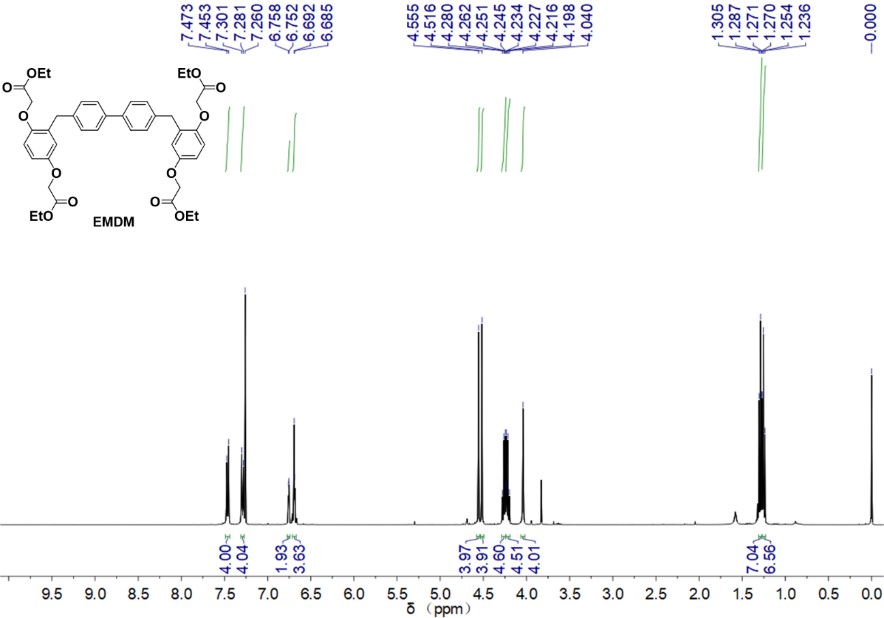


**Figure S9.** 1H NMR spectrum (400 MHz, CDCl3, 298 K) of EMDM.


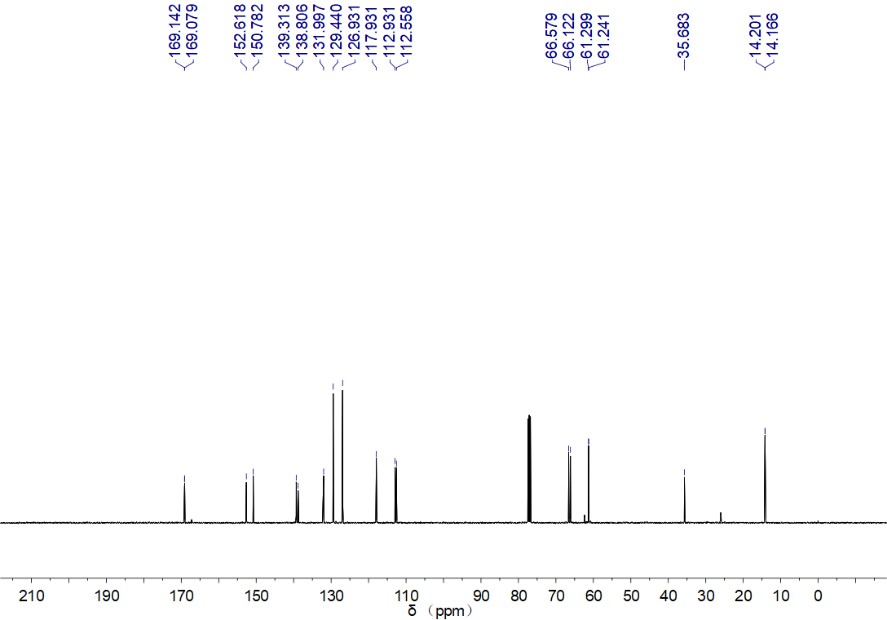


**Figure S10.** 13C NMR spectrum (101 MHz, CDCl3, 298 K) of EMDM.

**Synthesis of CMDM**: EMDM (2 g, 2.7 mmol) was added into a solution containing EtOH (35 mL) and NaOH solution (40% wt, 35 mL). After being refluxed for 24 h, EtOH was removed under reduced pressure. Hydrochloric acid was added to the water phase and the white precipitation was collected as product CMDM by filtration (1.3 g, 77%). 1H NMR (400 MHz, DMSO-*d6*, 298 K): δ 12.94 (s, 4H), 7.49 (s, 4H), 7.33 (s, 4H), 6.91-6.57 (m, 6H), 4.62 (s, 4H),

4.55 (s, 4H), 3.94 (s, 4H). 13C NMR (101 MHz, DMSO-*d6*, 298 K): δ 170.89, 170.81, 152.42,

150.38, 140.17, 138.21, 131.25, 129.79, 126.86, 117.64, 113.29, 112.41, 66.06, 65.40, 35.33.


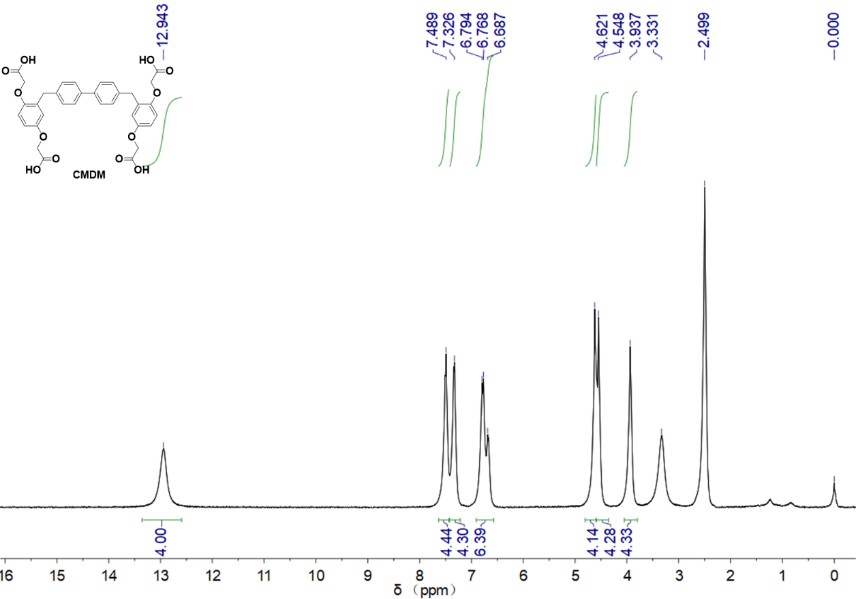


**Figure S11.** 1H NMR spectrum (400 MHz, DMSO-*d6*, 298 K) of CMDM.


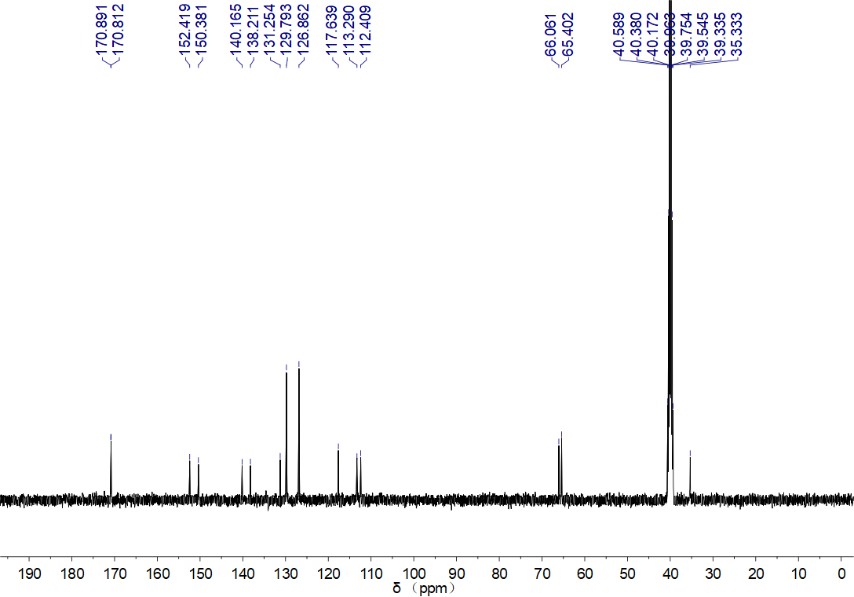


**Figure S12.** 13C NMR spectrum (101 MHz, DMSO-*d6*, 298 K) of CMDM.

**Synthesis of M**: CMDM (378.1 mg, 0.6 mmol) and sodium hydroxide (96 mg, 2.4 mmol) were dissolved in ultrapure water (5 mL). After stirring the reaction mixture for 20 min, M (420.3 mg, 97%) was obtained as a white solid by removing water under reduced pressure. 1H NMR (300 MHz, D2O, 298 K): δ 7.41 (d, *J* = 8.2 Hz, 4H), 7.29 (d, *J* = 8.3 Hz, 4H), 6.84 (d, *J* = 2.8

Hz, 2H), 6.77 (m, 4H), 4.36 (s, 4H), 4.32 (s, 4H), 3.99 (s, 4H). 13C NMR (101 MHz, D2O, 298

K): δ 177.39, 176.95, 151.99, 150.54, 139.96, 137.80, 131.37, 129.10, 126.61, 117.29, 113.57,

112.54, 68.15, 67.24, 35.21. HRMS (m/z): [M-4Na++3H+]− calcd for C34H29O12−, 629.1653, found 629.1699.


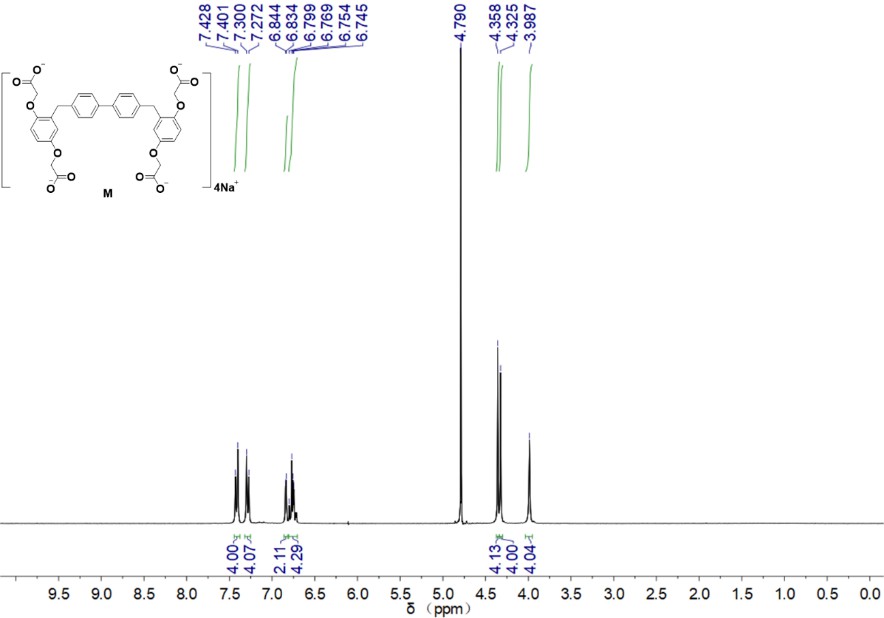


**Figure S13.** 1H NMR spectrum (300 MHz, D2O, 298 K) of M.


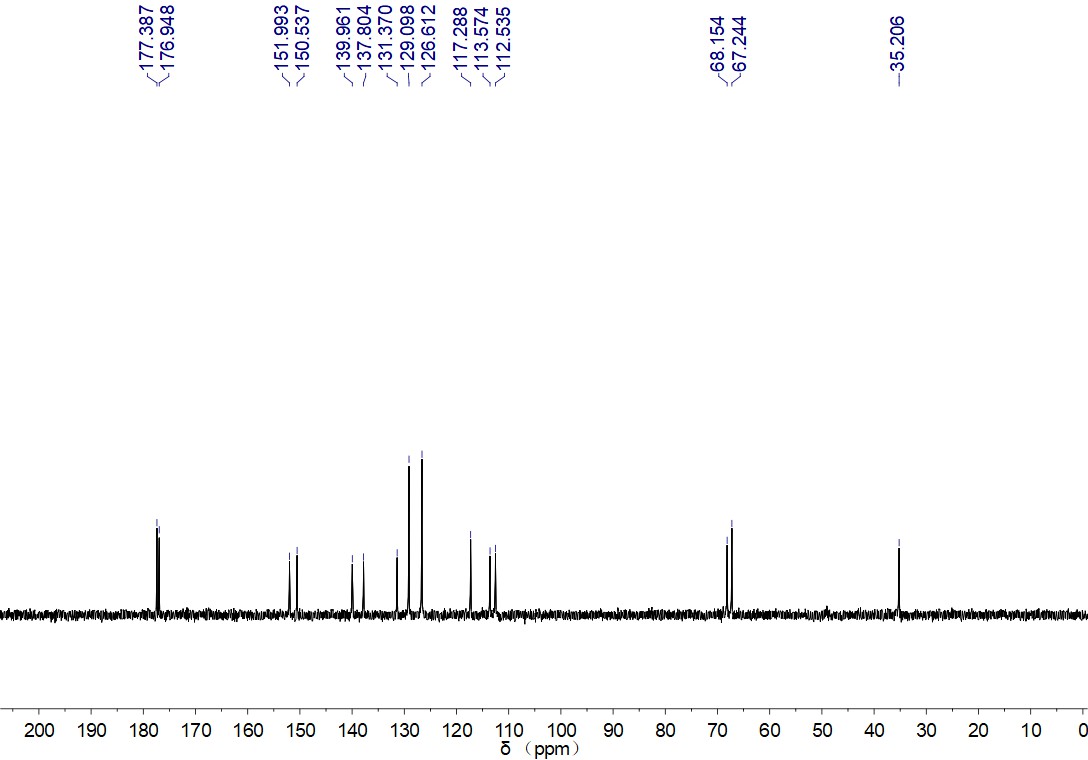


**Figure S14.** 13C NMR spectrum (101 MHz, D2O, 298 K) of M.

Intens. x104

1-

629.1699

-MS, 3.0min #177

1.5

1.0

1-

630.1725

0.5

1-

631.1750

637.6493

0.0

615 620 625 630 635 640 645 m/z

**Figure S15.** HRMS spectrum of M: [M-4Na++3H+]− calcd for C34H29O12− 629.1653, found

629.1699.

**2.3 Synthesis of diquat**S3

**Scheme S3.** Synthetic route to diquat.

2,2’-Bipyridyl (468.5 mg, 3.0 mmol) was added in 1,2-dibromoethane (20 mL) in a flask and then refluxed for 15 h, during which a precipitate was accumulated. The reaction was cooled to room temperature. The solid was filtered and washed with dichloromethane, then dried under vacuum to produce a brown product (778.9 mg, 75%). 1H NMR (300 MHz, D2O, 298 K): δ

9.21 (d, *J* = 6.1 Hz, 2H), 9.02-8.85 (m, 4H), 8.38 (m, 2H), 5.35 (s, 4H).


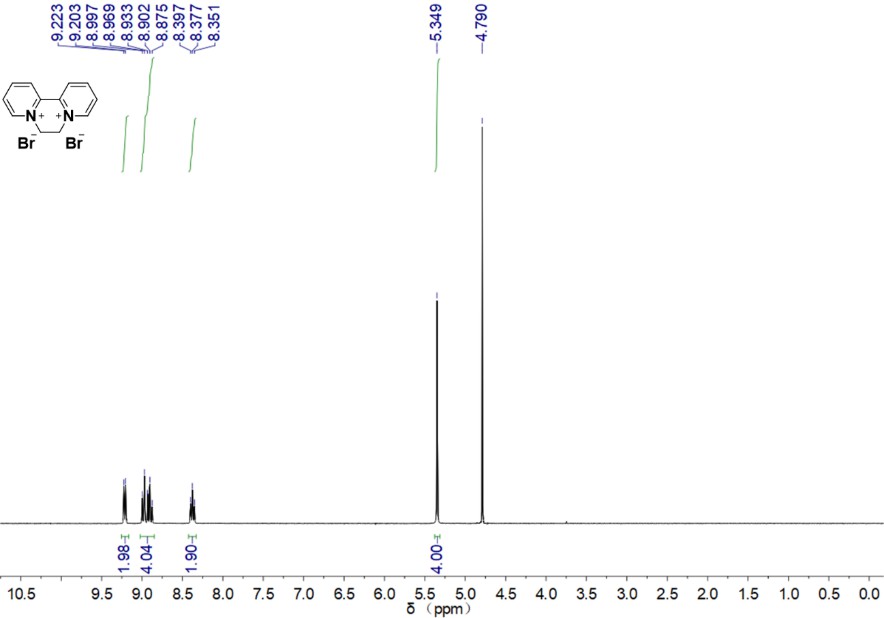


**Figure S16.** 1H NMR spectrum (300 MHz, D2O, 298 K) of diquat.

**2.4 Synthesis of WBpP6-AgNPs**

WBpP6-AgNPs was prepared according to the protocol previously reported by our group.S4

AgNO3 (10.0 mM, 207 µL) was added to ultrapure water (10 mL), followed by the addition of

WBpP6 solution (10.0 mM, 15 µL) under continuous stirring. Then freshly prepared NaBH4

solution (0.1 M, 100 µL) was added to the mixture solution. The reaction was further allowed for 30 min to afford the yellow WBpP6-AgNPs colloidal solution.

**2.5 Synthesis of M-AgNPs**

AgNO3 (10.0 mM, 207 µL) was added to ultrapure water (10 mL), followed by the addition of M solution (10.0 mM, 15 µL) under continuous stirring. Then freshly prepared NaBH4 solution (0.1 M, 100 µL) was added to the mixture solution. The reaction was further allowed for 30 min to afford the yellow M-AgNPs colloidal solution.

**3. Catalysis method**

**3.1 Catalytic reduction of the aromatic nitro group by WBpP6-AgNPs**

The catalytic activity of WBpP6-AgNPs for the reduction of the aromatic nitro group was investigated using 4-nitroaniline (*p*-NA) as a model compound. *P*-NA (110 µL, 3.0 mM), ultrapure water (2940 µL) and freshly prepared NaBH4 (150 µL, 0.1 M) were added to a standard quartz cuvette with 1 cm path length. Then WBpP6-AgNPs (50 µL, 86.7 µg/mL) were added into the mixture, and the UV-vis spectra were recorded every 2 minutes in the range of

200-800 nm. The reaction conditions for catalytic reduction of other aromatic nitro compounds are the same as those for *p*-NA.

**3.2 The stability of WBpP6-AgNPs for repeated catalytic cycles**

*p*-NA (110 µL, 3.0 mM) and fresh NaBH4 (25 µL, 0.1 M) were added to the above mixture. The timer was restarted immediately, and the UV-vis spectra were recorded in the same range and time interval. The procedure was repeated four times.

**4. Detection method**

Different concentrations of diquat solution (200 µL) were added to WBpP6-AgNPs colloid solution (1.8 mL); thereby, the mixture solutions of diquat (0-8.0 µM) were obtained, respectively. After balancing for 30 min, the diquat-induced aggregation of WBpP6-AgNPs was monitored by UV-vis spectroscopy.

**5. Supplementary data**

**5.1 Characterization of WBpP6-AgNPs**


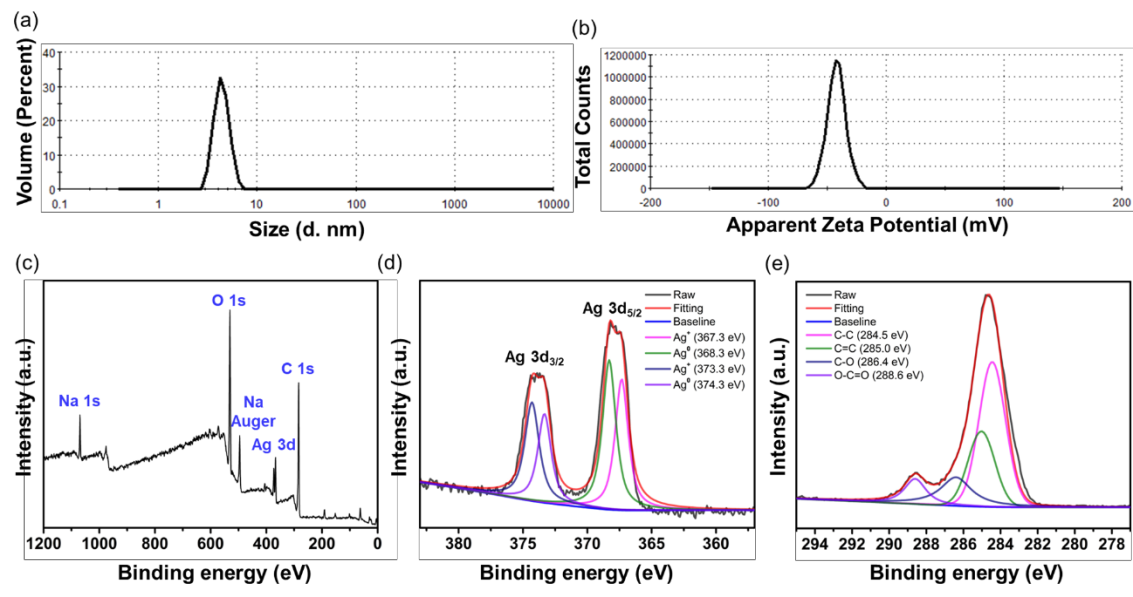


**Figure S17.** Characterization of WBpP6-AgNPs: (a) Distributions of hydrodynamic diameter according to dynamic light scattering (hydrodynamic diameter = ~4.4 nm); (b) Zeta potential distribution (-47.3 mV); The XPS survey spectrum of WBpP6-AgNPs (c); The high-resolution XPS of Ag 3d (d), and C 1s (e) in WBpP6-AgNPs.

**5.2 Characterization of M-AgNPs**


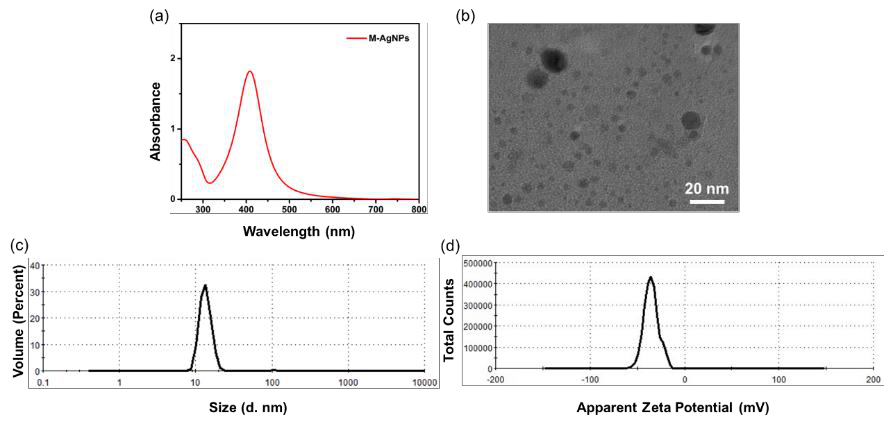


**Figure S18.** Characterization of M-AgNPs: (a) UV-vis spectrum. (b) TEM image. (c) Distributions of hydrodynamic diameter according to dynamic light scattering (hydrodynamic diameter = ~13.52 nm), and (d) zeta potential distribution (-35.0 mV).

**5.3 X-ray photoelectron spectrum of WBpP6**


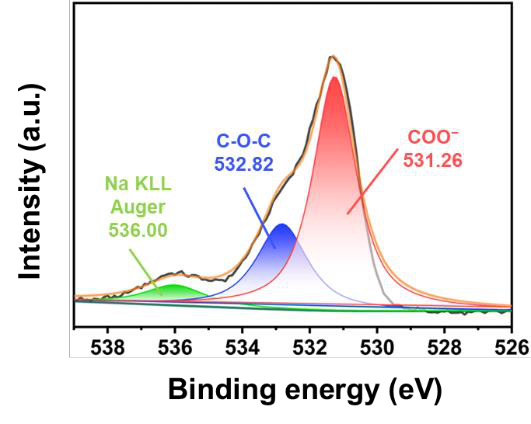


**Figure S19.** O 1s XPS spectra of WBpP6.

**5.4 The stability of WBpP6-AgNPs**


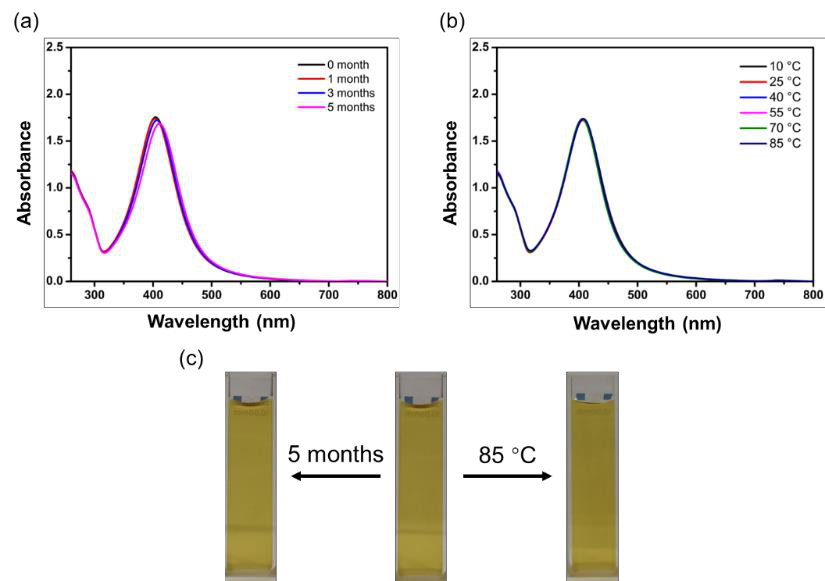


**Figure S20.** (a) Time-dependent UV-vis spectra changes of WBpP6-AgNPs solution. (b) Temperature-dependent UV-vis spectra changes of WBpP6-AgNPs solution. (c) Photographs of WBpP6-AgNPs solution: 5 months after preparation (left), freshly prepared (middle), after treated at high temperature (right).

**5.5 Catalytic Reduction of *p*-NA by different contents of WBpP6-AgNPs**


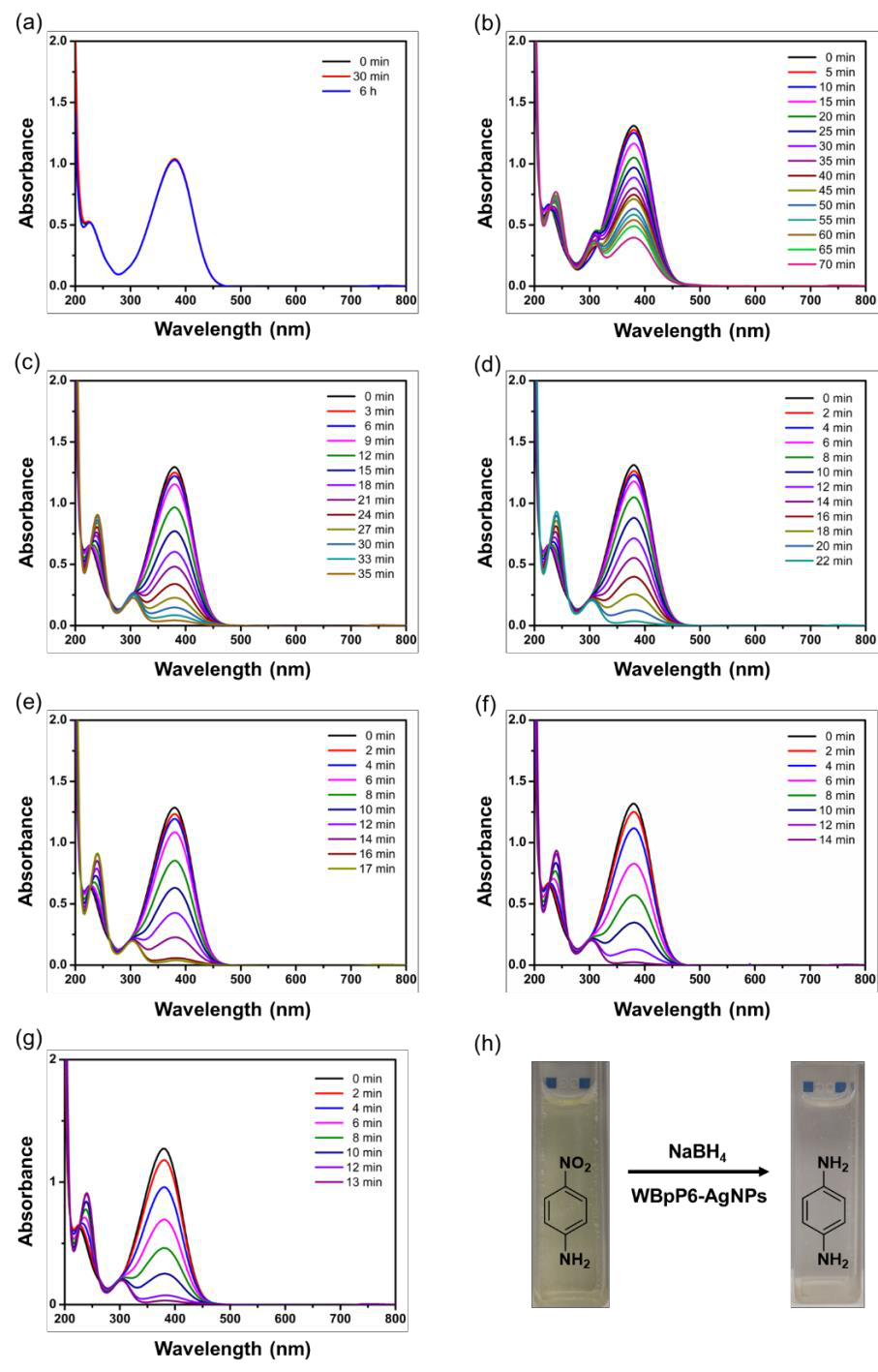


**Figure S21.** UV-vis spectra of *p*-NA solution reduced by NaBH4 with different contents of WBpP6-AgNPs: (a) 0 µL; (b) 10 µL; (c) 20 µL; (d) 30 µL; (e) 40 µL; (f) 50 µL; (g) 60 µL, and (h) the color of reaction system before and after catalytic reduction.

**5.6 Catalytic reduction of aromatic nitro compounds by WBpP6-AgNPs**


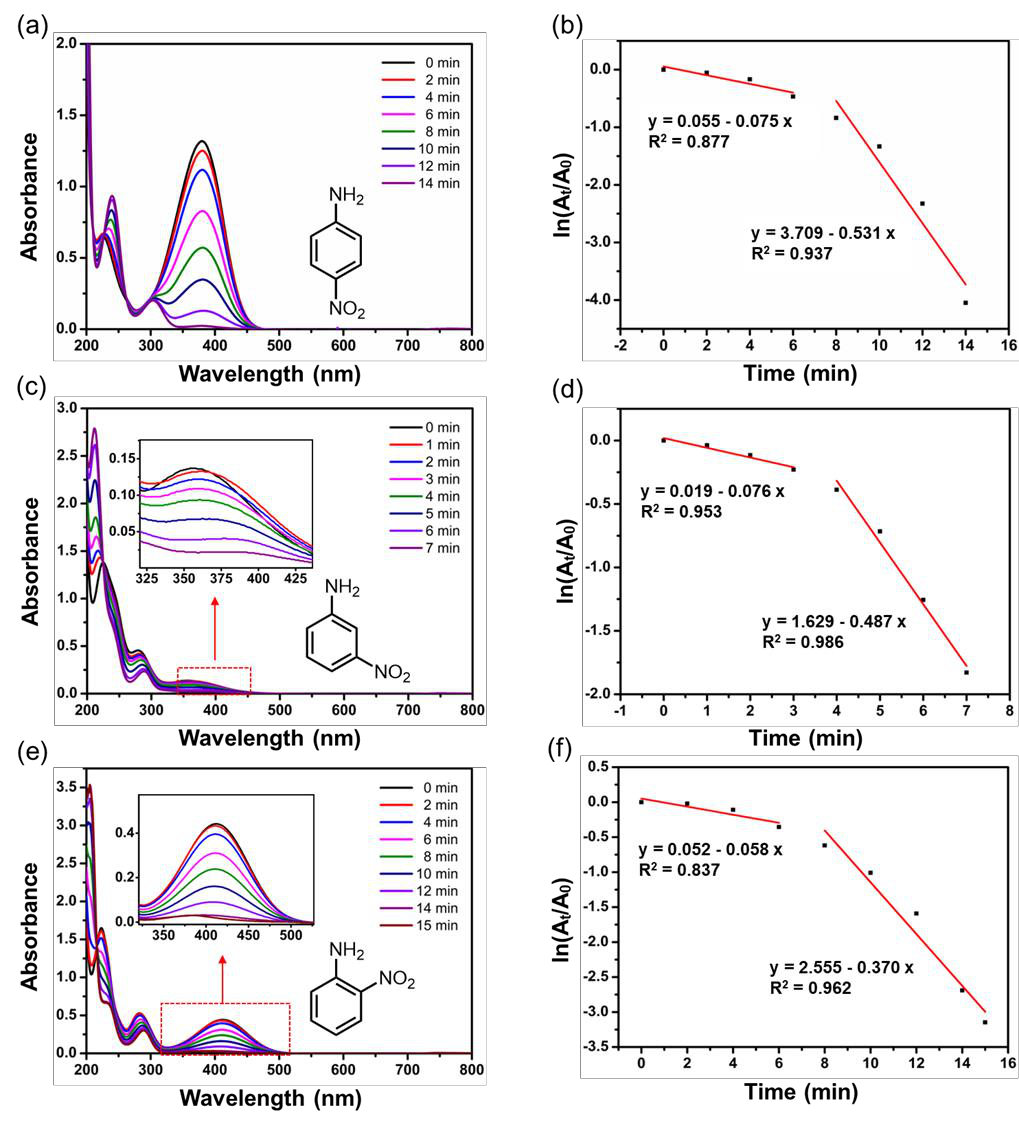


**Figure S22.** Catalysis of nitroaniline by WBpP6-AgNPs with NaBH4: time-dependent UV-vis spectra changes for (a) *p*-NA, (c) *m*-nitroaniline (*m*-NA) and (e) *o*-nitroaniline (*o*-NA). The ln(*At*/*A0*) versus time for the catalytic reduction of (b) *p*-NA, (d) *m*-NA, and (f) *o*-NA.


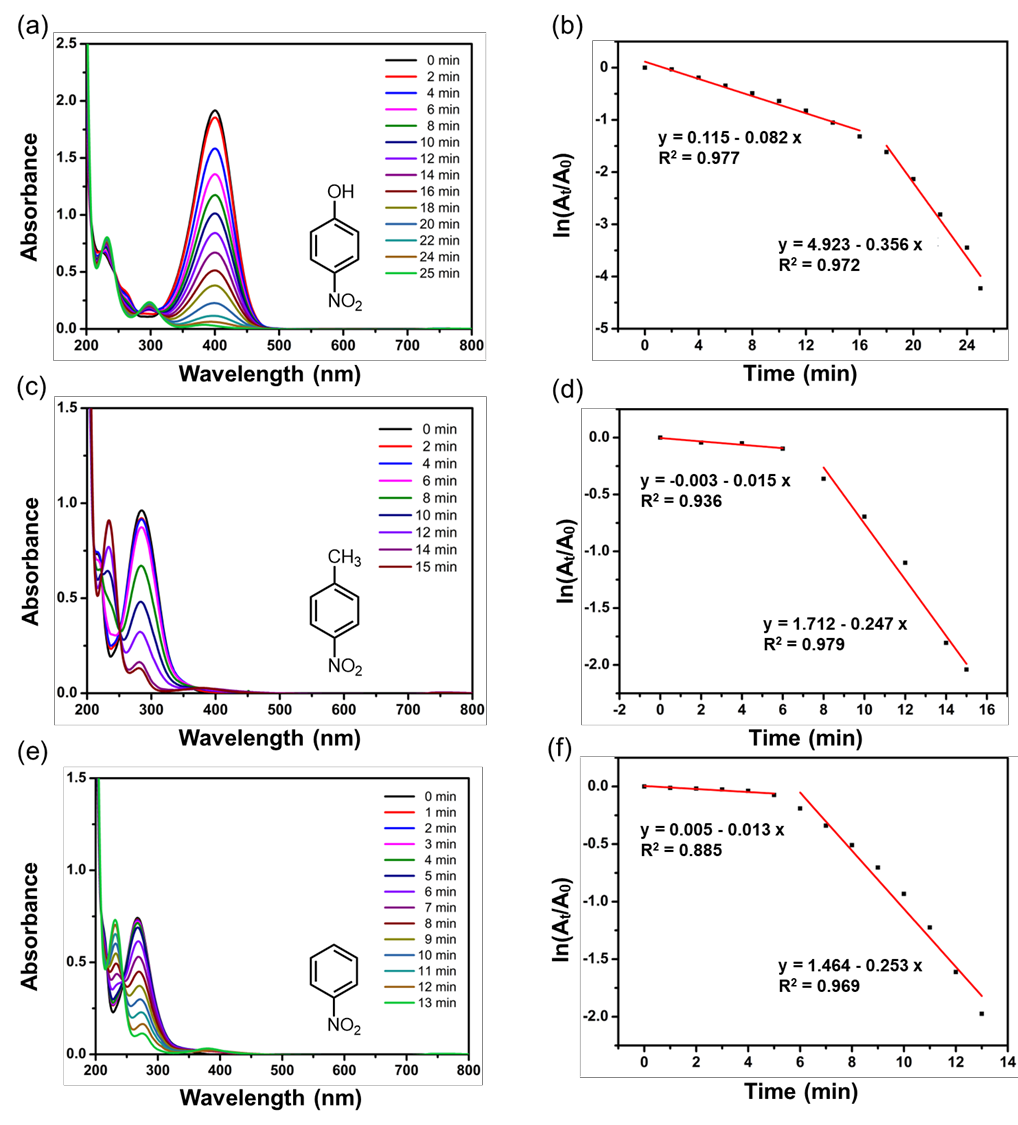


**Figure S23.** Catalysis of aromatic nitro compounds by WBpP6-AgNPs with NaBH4: time- dependent UV-vis spectra changes for (a) *p*-nitrophenol (*p*-NP), (c) *p*-nitrotoluene (*p*-NT) and (e) nitrobenzene (NB). The ln(*At*/*A0*) versus time for the catalytic reduction of (b) *p*-NP, (d) *p*- NT, and (f) NB.


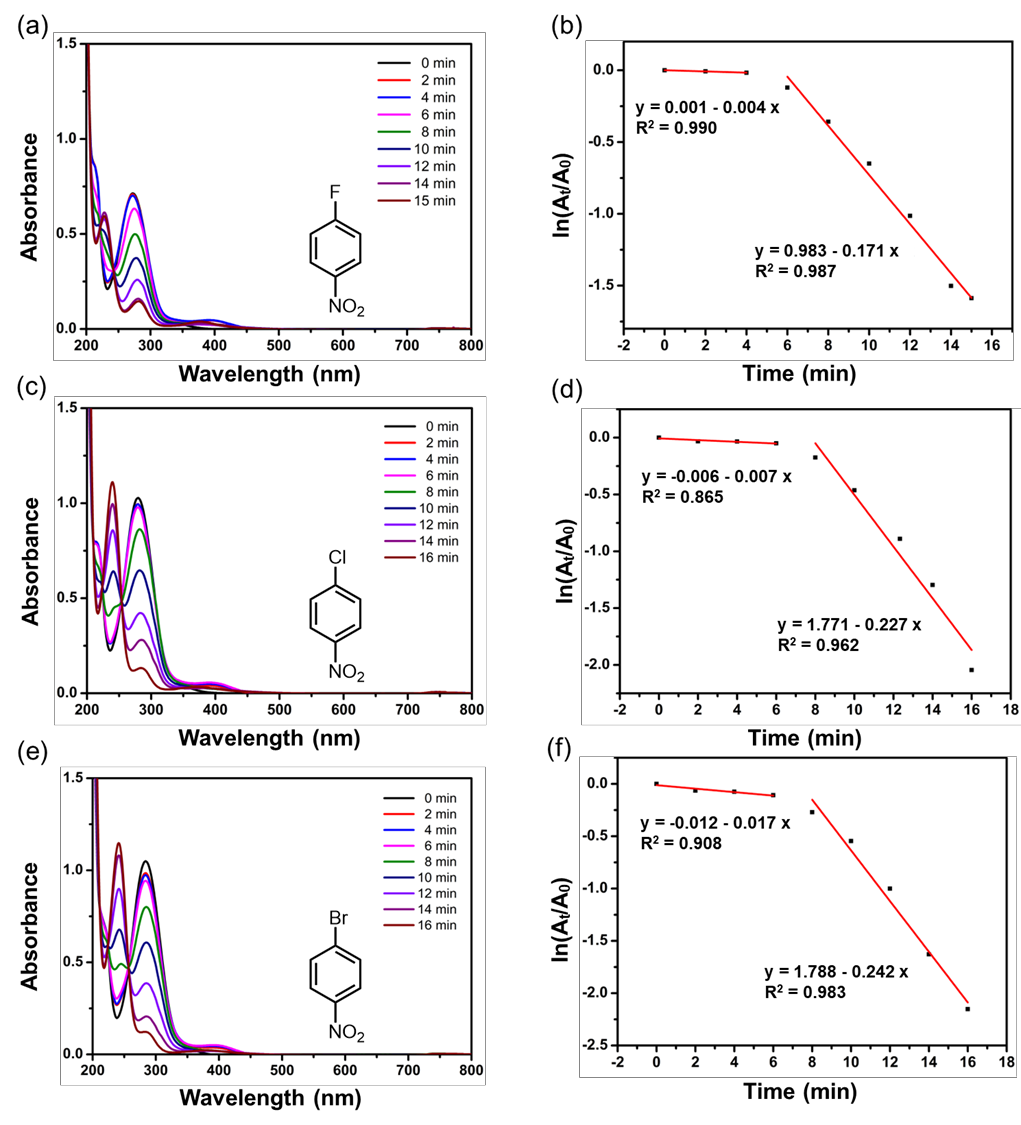


**Figure S24.** Catalysis of aromatic nitro compounds by WBpP6-AgNPs with NaBH4: time- dependent UV-vis spectra changes for (a) *p*-fluoronitrobenzene (*p*-FNB), (c) *p*- chloronitrobenzene (*p*-CNB) and (e) *p*-bromonitrobenzene (*p*-BNB). The ln(*At*/*A0*) versus time for the catalytic reduction of (b) *p*-FNB, (d) *p*-CNB, and (f) *p*-BNB.


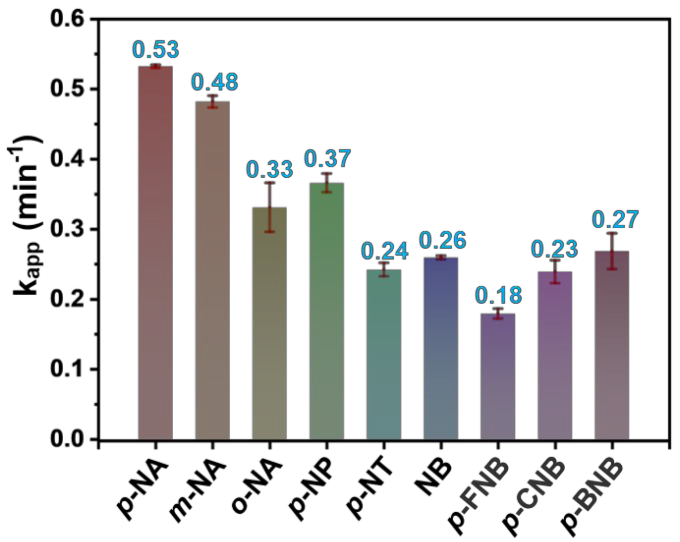


**Figure S25.** The rate constants for the reduction of aromatic nitro compounds using WBpP6- AgNPs as a catalyst.

**5.7 Catalytic reduction of *p*-NA by M-AgNPs**


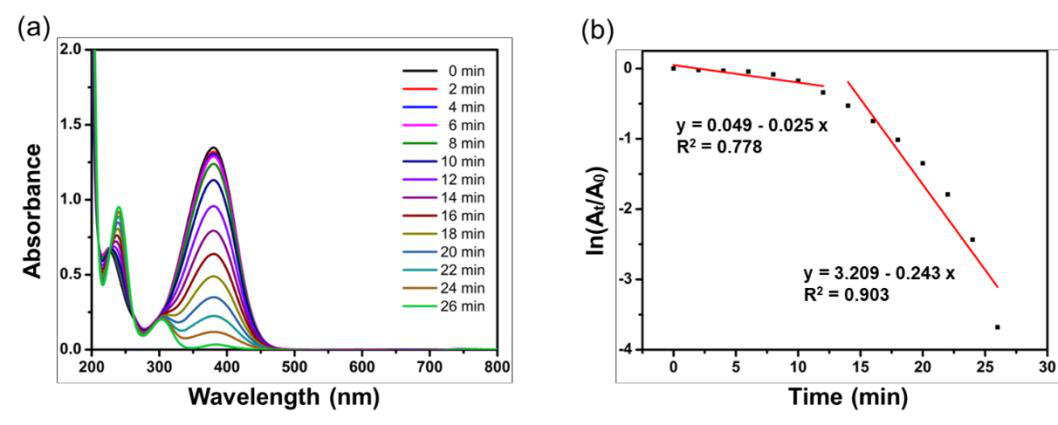


**Figure S26.** Time-dependent UV-vis spectra of *p*-NA solution reduced by NaBH4 using WBpP6-AgNPs as a catalyst and the corresponding ln(*At*/*A0*) versus time for the catalytic reduction.

**5.8 The intermolecular charge-transfer (ICT) interaction between WBpP6 and diquat**


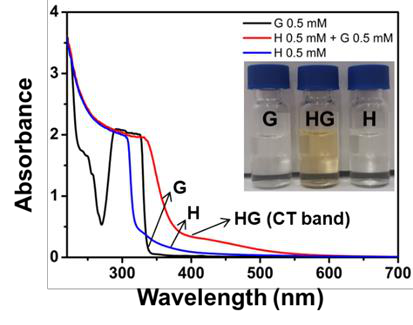


**Figure S27.** UV-vis spectra (H2O): (G) diquat (0.5 mM); (H) WBpP6 (0.5 mM); (HG) WBpP6 (0.5 mM) and diquat (0.5 mM). The inserted photograph shows the deepening color upon the mixtures of host–guest donor-acceptor (D-A) pairs due to the ICT interaction between WBpP6 and diquat.

**5.9 The control experiments of diquat detection**


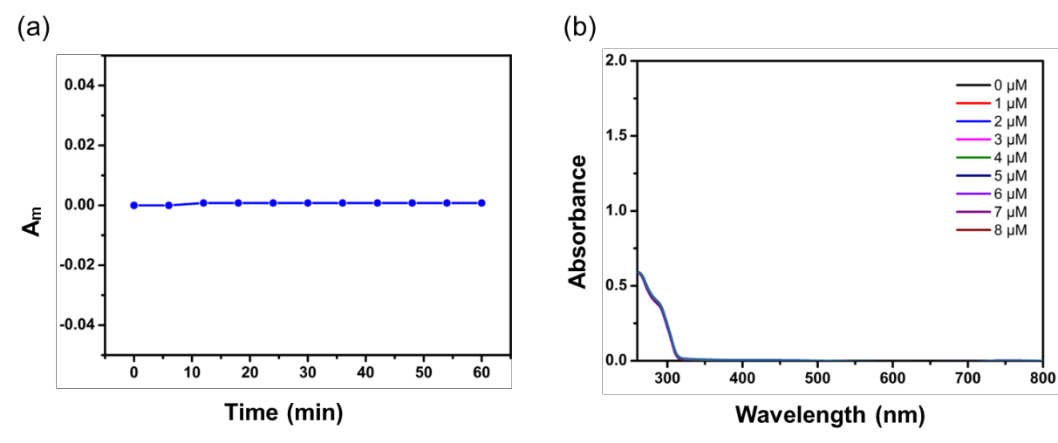


**Figure S28.** (a) Relationship between decreasing absorbance of WBpP6-AgNPs at 405 nm (*Am*) with time when 20 μL ultrapure water was added into WBpP6-AgNPs. (b) UV-vis spectra of WBpP6 mixed with different concentrations of diquat.

**6. References**

S1. J. Yang, D. Dai, L. Ma, Y.-W. Yang, *Chin. Chem. Lett.* **2021**, *32*, 729.

S2. Y. Zhang, Z. Li, S. Meng, A. Dong, Y.-W. Yang, *Chem. Commun.* **2022**, *58*, 649.

S3. Z. Zhang, J. Huang, K. Gallagher (UChicago Argonne, LLC), *US20180191012*, **2018**.

S4. X. Wang, Z.-J. Liu, E. H. Hill, Y. Zheng, G. Guo, Y. Wang, P. S. Weiss, J. Yu, Y.-W. Yang,

*Matter* **2019**, *1*, 848.
